# Integration of clinical phenoms and metabolomics facilitates precision medicine for lung cancer

**DOI:** 10.1007/s10565-024-09861-w

**Published:** 2024-05-01

**Authors:** Furong Yan, Chanjuan Liu, Dongli Song, Yiming Zeng, Yanxia Zhan, Xibing Zhuang, Tiankui Qiao, Duojiao Wu, Yunfeng Cheng, Hao Chen

**Affiliations:** 1 Center for Tumor Diagnosis & Therapy, Jinshan Hospital, Fudan University, Shanghai, 201508 China; 2 Department of Pulmonary and Critical Care Medicine, Zhongshan Hospital, Fudan University, Shanghai, 200032 China; 3 Center of Molecular Diagnosis and Therapy, The Second Affiliated Hospital of Fujian Medical University, Quanzhou, 362000 Fujian China; 4 Shanghai Institute of Clinical Bioinformatics, Shanghai, 200032 China; 5 Institute of Clinical Science, Zhongshan Hospital, Fudan University, Shanghai, 200032 China; 6 Department of Hematology, Zhongshan Hospital, Fudan University, 180 Fenglin Rd, Shanghai, 200032 China; 7 Department of Thoracic Surgery, Zhongshan-Xuhui Hospital, Fudan University, 366 North Longchuan Rd, Shanghai, 200237 China; 8 Department of Hematology, Xiang’an Hospital, Xiamen University School of Medicine, Xiamen, 361101 China

**Keywords:** Lipidomics, Polar metabolites, Lung cancer, Clinical phenoms, Trans-omics

## Abstract

Lung cancer is a common malignancy that is frequently associated with systemic metabolic disorders. Early detection is pivotal to survival improvement. Although blood biomarkers have been used in its early diagnosis, missed diagnosis and misdiagnosis still exist due to the heterogeneity of lung cancer. Integration of multiple biomarkers or trans-omics results can improve the accuracy and reliability for lung cancer diagnosis. As metabolic reprogramming is a hallmark of lung cancer, metabolites, specifically lipids might be useful for lung cancer detection, yet systematic characterizations of metabolites in lung cancer are still incipient. The present study profiled the polar metabolome and lipidome in the plasma of lung cancer patients to construct an inclusive metabolomic atlas of lung cancer. A comprehensive analysis of lung cancer was also conducted combining metabolomics with clinical phenotypes. Furthermore, the differences in plasma lipid metabolites were compared and analyzed among different lung cancer subtypes. Alcohols, amides, and peptide metabolites were significantly increased in lung cancer, while carboxylic acids, hydrocarbons, and fatty acids were remarkably decreased. Lipid profiling revealed a significant increase in plasma levels of CER, PE, SM, and TAG in individuals with lung cancer as compared to those in healthy controls. Correlation analysis confirmed the association between a panel of metabolites and TAGs. Clinical trans-omics studies elucidated the complex correlations between lipidomic data and clinical phenotypes. The present study emphasized the clinical importance of lipidomics in lung cancer, which involves the correlation between metabolites and the expressions of other omics, ultimately influencing clinical phenotypes. This novel trans-omics network approach would facilitate the development of precision therapy for lung cancer.

**Graphical Abstract:**

1. Integrating multiple biomarkers or trans-omics results improves diagnostic accuracy and reliability in heterogeneous lung cancer.

2. Metabolomics and lipidomics, along with clinical phenotypes, construct a comprehensive metabolic profile of lung cancer patients.

3. TAG expression shows strong positive correlation with polar metabolites, potentially impacting clinical phenotypic changes in lung cancer patients.

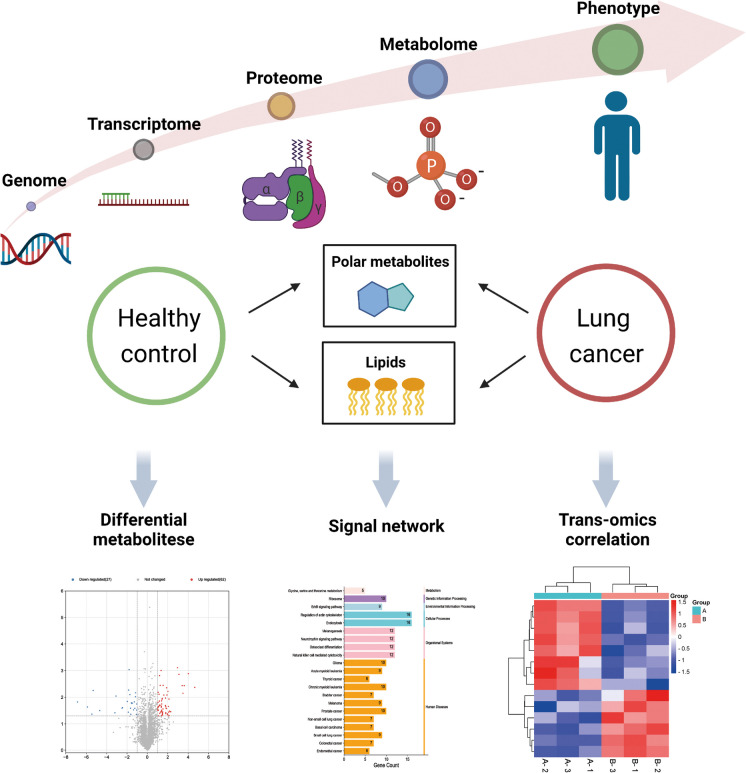

**Supplementary Information:**

The online version contains supplementary material available at 10.1007/s10565-024-09861-w.

## Introduction

Among cancers related to cigarette smoking, environmental pollution, toxic substances, or chronic diseases, lung cancer is the most common malignancy (Bray et al. 2018;Fang et al. 2011). Numerous targeted therapies have been developed as the first-line treatment for lung cancer. However, the sensitivity to these therapies varies widely, and cancer recurs often after treatment, largely due to the intratumoral and intertumoral heterogeneity of metabolic disturbances. Metabolic regulation of tumor cells mainly includes tumor cell intrinsic metabolism, interactions between cancer and non-cancer cells, tumor location and heterogeneity, and systemic metabolism(Elia and Haigis 2021). The existence of systemic metabolic disorders in lung cancer could potentially serve as a foundation for the development of biomarkers used in diagnosis and prognosis. The progression of technology has led to an increased scientific significance of metabolomics in comprehending the molecular mechanisms of lung cancer(Chen et al. 2019; Singh et al. 2022; Zhang et al. 2020).

Based on qualitative and quantitative analyses of the metabolites in cells, tissues, and whole organisms, metabolomics focuses on polar metabolites (water solubility) such as amino acids, carbohydrates, and organic acids. Lipidomics is an emerging independent branch of metabolomics, with a clear focus on the comprehensive identification and quantification of lipids (Han 2016; Liu et al. 2021a, b). Systemic metabolic disorders in lung cancer patients include abnormalities of polar metabolites and lipid metabolism. The abnormality of plasma-free amino acid profiles or glucose-related metabolites, as well as the difference of glucose metabolism in lung cancer cells between lung cancer and healthy, has signified the values of amino acids or glucose metabolism diversity, i.e., pathways of glycolysis and gluconeogenesis, for early diagnosis of lung cancer (Ding et al. 2019; Liu et al. 2021a, b; Louis et al. 2016; Shingyoji et al. 2013; Singh et al. 2022). Lipid classes of plasma include phosphatidylcholines (PC), phosphatidylethanolamine (PE), lysophosphatidylcholine (LPC), lysophosphatidylethanolamine (LPE), sphingomyelin (SM), triacylglycerol (TAG), diacylglycerol (DAG), and cholesteryl ester (CE). Alterations in circulating lipid profiles have been identified as potential plasma lipid markers for the early detection of lung cancer and for distinguishing between different cancer subtypes, including lung adenocarcinoma (ADC), squamous cell carcinomas (SCC), and small cell lung cancer (SCLC) (Ros-Mazurczyk et al. 2017; Yu et al. 2018; Zhang et al. 2020; Zhu et al. 2020). It has been reported that plasma levels of LPC 16:0, 18:0, 18:1 and 18:2, PCs, PEs, and some phospholipids in lung cancer patients were significantly different from healthy (Chen et al. 2018; Marien et al. 2015; Yu et al. 2018; Zhu et al. 2020).

The metabolic disturbance is caused by dysfunctional activation of certain enzymes and ultimately affect signaling pathways (Wang et al. 2022). Integrated metabolomics and lipidomics offer a holistic view of the metabolic landscape, facilitating a comprehensive network analysis to uncover pivotal metabolic factors in diseases. This approach has been applied for investigating potential metabolism-associated biomarkers of malignant pleural effusion in late-stage lung cancer with metastasis (Yang et al. 2020a, b). Lipidomic and metabolomic profiles of lung cancer tissues and para-cancer normal tissues also revealed a clear association between central carbon metabolic pathways with the disorder of lipid metabolism related to glycerophospholipids, sphingolipids, and cholesteryl esters (Cifkova et al. 2022). Using clinical lipidomics, we can discover the correlation and regulation between various lipids and clinical phenotypes (Zhu et al. 2020). Using this tool, it has been found that plasma lipids of lung cancer patients are significantly correlated with clinical phenomes such as gender, age, stage, metastatic status, nutritional status, and severity of clinical symptoms (Zhu et al. 2020).

Although studies have demonstrated mighty metabolic dysfunctions in lung cancer, large-scale researches connecting multiple metabolomic with clinical phenoms of lung cancer, which is crucial for a better understanding of the metabolic characteristics of lung cancer, are still lacking. This study aimed to investigate the specificity and correlation between plasma metabolic profiles and disease manifestations in lung cancer. By combining metabolomics, lipidomics, and clinical phenotypes associated with the disease, the study sought to identify potential metabolic targets that could potentially serve as prospective markers for lung cancer. Furthermore, the study would pave the path to explore the clinical phenotypically dependent metabolic pathways and the underlying mechanisms in lung cancer subtypes, including ADC, SCC, and SCLC.

## Materials and methods

### Chemical agents

All laboratory standards, chemicals, and reagents met the needs of mass spectrometry. The internal standard for polar metabolite detection was tridecanoic acid (Sigma-Aldrich, St. Louis, MO, USA). Internal Standards Kit for Lipidyzer™ Platform (SCIEX, Darmstadt, Germany) was used for lipids detection. Fisher Chemical (Waltham, MA, USA) supplied methanol, water, acetonitrile, isopropanol, and chloroform. The methoxyamine hydrochloride, ammonium hydroxide solution, pyridine, acetone, ammonium acetate, and N-methyl-N-(trimethylsilyl) trifluoroacetamide were obtained from Sigma-Aldrich (St. Louis, MO, USA).

### Patient cohort

Pathologically confirmed lung cancer (ADC, SCC, and SCLC) patients were recruited from June 2018 to December 2020. In accordance with the 8th edition of the TNM classification for lung cancer, the stage and severity were delineated (Goldstraw et al. 2016). Healthy controls (HC) were consisted of age- and gender- matched adult volunteers enrolled during the same study period. Peripheral venous blood samples were collected upon admission, along with their clinical records. Samples of 111 lung cancer patients and 111 volunteers were processed for polar metabolite analysis, and 204 lung cancer patients and 204 volunteers for lipid analysis. Among these lung cancer patients, pathological classification was confirmed in 33 patients. These patients included 20 adenocarcinoma (ADC), 4 squamous cell carcinomas (SCC), and 9 small cell lung cancer (SCLC). The study protocol obtained the approval of the Institutional Review Board of the hospital (# IEC-2020-S34). Written informed consents were obtained from all participants upon enrollment.

### Digital evaluation score system

DESS (Digital Evaluation Score System) is a scoring index system employed to convert clinical descriptive information into clinical informatics (Zhang et al. 2020). A scoring system was used to evaluate each component, assigning scores of 0, 1, 2, or 4. A score of 4 denoted the most severe condition, while a score of 0 indicated a normal physiological state, as detailed in Supplementary Table 1. The DESS scores spanned from 0 to 1868, with higher scores indicating greater severity of the condition. In the current study, 467 clinical phenomes were scored and collected from lung cancer patients, including 54 from patient history, 64 from symptoms, 62 signs, 142 from clinical chemical measurements, 92 from image features, and 53 from pathology indexes.

### Polar metabolomics detection

Samples were kept at -80 °C until use and thawed overnight in a refrigerator prior to sample preparation. We used 100 μl of plasma from each sample for metabolites extraction. For quality control, equal aliquots of samples were combined. An internal standard of 5 μg/ml tridecanoic acid was added to the plasma samples in a 400 μl solution (ratio of volume, methanol: water = 4:1). After vigorous mixing at 37 °C for 30 s, the mixtures were gently shaken at 1200 rpm for 30 min. We centrifuged the mixtures at 14,000 rpm for 15 min at 4 °C and collected the supernatant. Supernatants were concentrated, frozen, and freeze-dried. Per manufacture’s two-step derivatization protocol, samples were resuspended in 50 μl of 20 mg/ml methoxyamine hydrochloride in pyridine. The tubes were vortexed, sonicated for one minute, and then incubated with shaking at 30 °C for 90 min to form methoxyamine derivatives. Subsequently, 40μl of N-methyl-N-(trimethylsilyl) trifluoroacetamide was added to the sample for silylation reaction and incubated in 37 °C for 30 min. For GC–MS analysis, the supernatants were collected after centrifugation at 14,000 rpm for 5 min. Untargeted metabolomics analysis employed gas chromatography mass spectrometry (GC–MS). Agilent 7890B GC and 5977B inert mass selective detector (MSD) system (Agilent Technologies, Santa Clara, CA, USA) were used. The raw data from GC–MS was performed using Agilent MassHunter Qualitative Analysis software (version 10.0, Agilent, CA, USA). The identification of metabolites was conducted by referencing the Agilent Fiehn database (Kind et al. 2009). To quantify the metabolite content in the sample, the following formula was employed: standard sample peak area divided by standard sample concentration equals sample peak area divided by sample concentration.

### Lipidomics detection

Twenty microliters, processed plasma, and reagents for quality control were maintained at -80 °C. Isopropanol was precooled at -20 °C, of which 350 μl isopropanol was precooled to 4 °C, and a 9 μl internal standard mixture was used for each sample. For improved protein precipitation, the solution was mixed for 1 min before being incubated at room temperature for 10 min. The samples were stored overnight at -20 °C. On the next day, the samples were centrifuged at 12,000 rpm for 20 min. The 200 μl supernatant was collected in a sample tube and stored at -80 °C for MS analysis. Plasma lipidomics was detected by AB SCIEX QTRAP 5500 liquid chromatography mass spectrometry (LC–MS)/MS system (Foster City, CA, USA). The extracted samples were introduced into a Waters Acquity UPLC BEH HILIC column (100 mm × 2.1 mm, 1.7 µm) coupled with a Waters Acquity UPLC BEH HILIC VanGuard Pre column (2.1 mm × 5 mm, 1.7um) for analysis. A phase was 95% acetonitrile (ratio of volume, acetonitrile: water = 95:5) contained 10 mmol/L ammonium acetate, and B phase was 50% acetonitrile (ratio of volume, acetonitrile: water = 50:50) containing with 10 mmol/L ammonium acetate. Ammonium hydroxide was incorporated into the B phase until it reached the same pH level as the A phase. The flow rate was set at 0.5 ml/minute. The gradient elution procedure involved the following steps: The B phase commenced at 0.1% and gradually increased to 20% over a period of 10 min, followed by a rapid increase to 98% between 10 to 11 min. The eluent was maintained at 98% B phase for 2 min before returning to the initial condition of 0.1% in 13.1 min. Subsequently, the system was held at 0.1% B phase for 16 min. The injection volumes for positive and negative electrospray ionization (ESI + and ESI-) modes were 2 and 5 μl, respectively, with N2 used as the dissolvent. The parameter settings were as follows: curtain gas at 35 psi, GS1 at 50 psi, GS2 at 60 psi, ion spray voltage at 5500 V, declustering potential at 80 V, entrance energy at 10 V, and collision energy at 50 V. Data were acquired using the Analyst software (version 1.7, SCIEX, MA, USA).

### Comprehensive analyses of multi-omic profiles

SIMCA 14.1 software (Umetrics, Umea, Sweden) was utilized for conducting principal component analysis (PCA) and orthogonal projection to latent structures discriminant analysis (OPLS-DA). To improve the differentiation between groups and gain a better understanding of the variables contributing to classification, supervised orthogonal projection to latent structures discriminant analysis (OPLS-DA) was employed. Subsequently, the software provided R2Y and Q2Y classification parameters, which were assessed for stability and predictive accuracy. The R2 and Q2 intercept values were obtained through 200 permutations, with lower Q2 intercept values indicating reduced risk of overfitting and increased reliability. The loading plot, generated using OPLS-DA, illustrated the contribution of variables towards the group differences. The heatmap analysis to examine the differences among the components was performed using MetaboAnalyst software 4.0 (www.metaboanalyst.ca). For enhanced analysis, the first principal component was computed as the variable importance projection (VIP), which helped prioritize the variables based on their significance. Metabolites with VIP values exceeding 1.0 were selected to identify the altered metabolites. Subsequently, Student's t-tests were conducted on the remaining variables with a significance threshold of *p* > 0.05, and metabolites without significant differences were removed. Additionally, the fold change (FC) was utilized to evaluate the variations in compound levels. The construction of receiver operating characteristic (ROC) curves involved plotting the true positive rate on the y-axis against the false positive rate on the x-axis. Correlations between polar metabolites and lipids were calculated using the Pearson correlation. The evaluation of trans-nodules between lipidomic profiles and clinical phenomes was conducted using the expression quantitative trait locus (eQTL) model.

### Statistical analysis

The mean ± SE for each group was calculated and compared. Statistical significance of differences between two groups or multiple groups was assessed using either Student's t-tests or one-way ANOVA tests. Statistical significance was affirmed when *p* < 0.05. The statistical analysis of the data obtained from the mass spectra was performed using SIMCA 14.1. The boxplot and heatmaps were constructed using the package ggplot2 in Rstudio. The volcano maps displayed notable changes in polar metabolites and lipids, indicating significant elevations or declines in lung cancer patients. Pie chart was plotted by http://www.bioinformatics.com.cn. Differential multi-omics metabolites contributing to lung cancer were identified using VIP value, FC values, and the corresponding *p* values. The lipid quantitative trait loci model modified from the eQTL model was used to investigate the correlation between lipid elements and clinical phenomes. Further, phenome-lipid pairs with significant *p* values were obtained using MatrixlQTL R package. Lipid levels in different lung cancer subtypes of ADC, SCC, and SCLC were separately calculated and then ranked to obtain the top 3 significantly changed lipids. We use Graph Pad Prism 7.04 (Graph Pad Software Inc., CA, USA) to measure the value of the specific alternations of differential lipids in ADC, SCC, or SCLC.

## Results

### Polar metabolites and lipid profiling of lung cancer

**Fig. 1:**
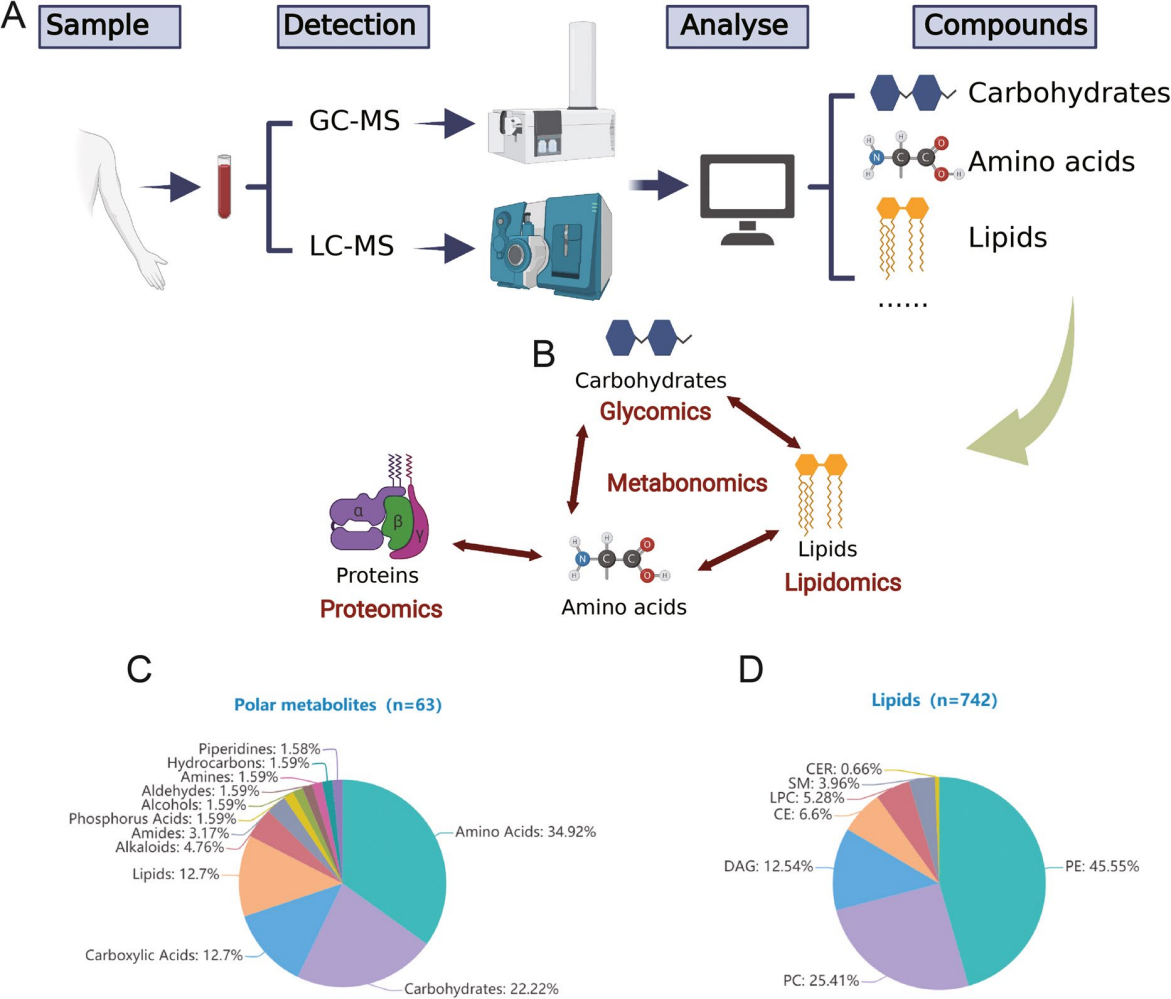
Overview of polar metabolome and lipidome detection in lung cancer. A schematic illustration showing the combined analysis using multi-omic metabolites data in the study for lung cancer precision medicine. In summary, blood samples were collected and subjected to detection and identification using LC–MS and GC–MS techniques. Subsequently, a multi-omics correlation analysis was conducted to explore the relationships between the identified metabolites. **A** Omics such as glycomics, metabonomics, lipidomics and proteomics are interconnected **B** Pie charts showing the numbers and proportions of annotated polar metabolites **C** and lipids **D** identified in present study. Abbreviations: GC–MS: gas chromatography mass spectrometry; LC–MS: liquid chromatography-mass spectrometry; PC: phosphatidylcholines; PE: phosphatidylethanolamine; LPC: lysophosphatidylcholine; LPE: lysophosphatidylethanolamine; SM: sphingomyelin; TAG: triacylglycerol; DAG: diacylglycerol; CE: cholesteryl ester

To comprehensively profile the lung cancer metabolome, the plasma of lung cancer patients and HC were detected for polar metabolites and lipids (Fig. 1, panel A). Internal standards and quality control samples were used for quality assurance. Multiple metabolites of polar metabolites (such as carbohydrates and amino acids) closely related to lipids, and together with protein metabolism, constitute the human metabolic map (Fig. 1 panel B). A total of 63 polar metabolites and 742 lipids were annotated (Fig. 1, panels C and D).

### The polar metabolomic landscape of lung cancer

**Fig. 2:**
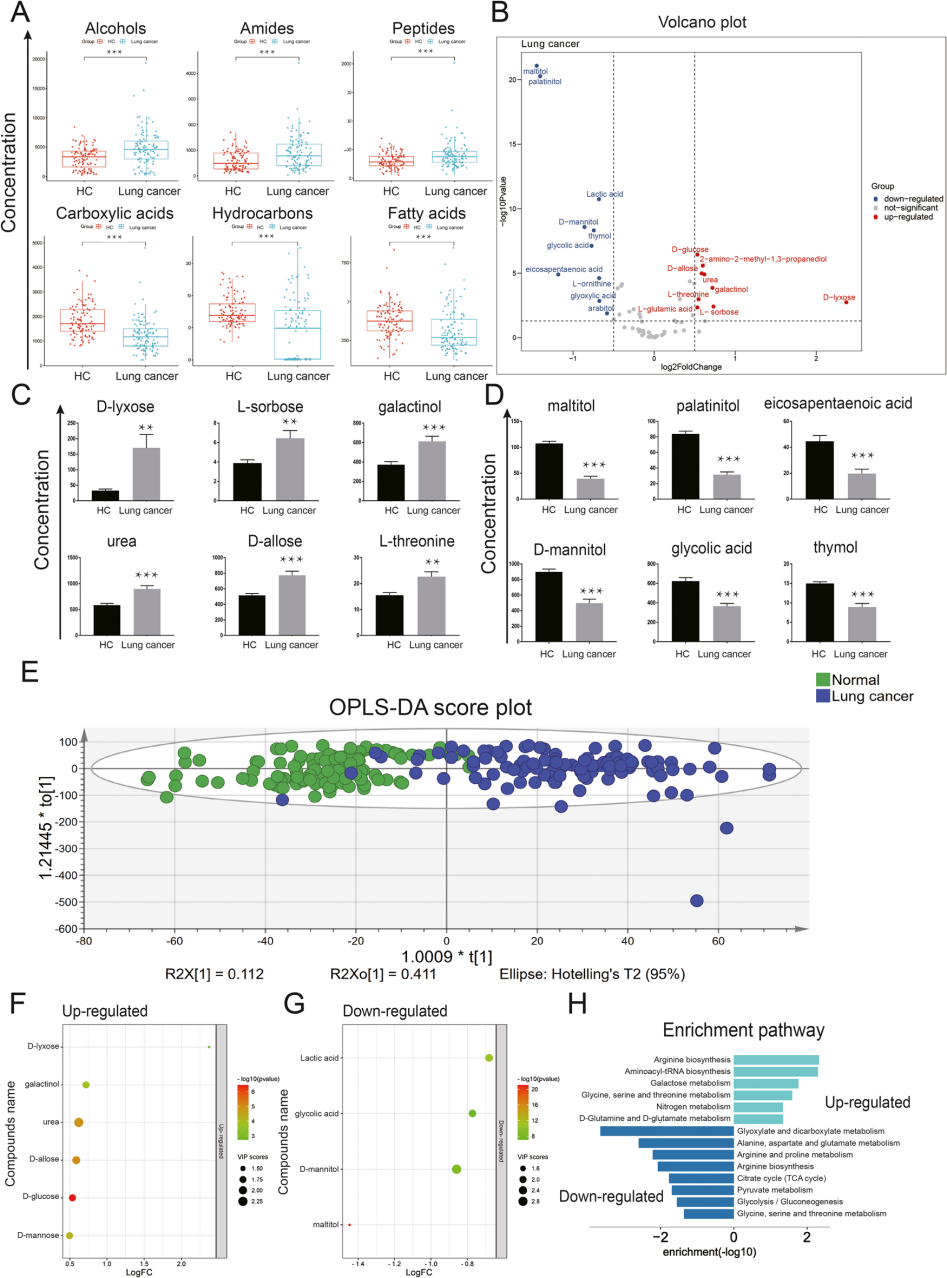
The metabolomic landscape of lung cancer. **A** Changes in metabolic profiles in plasma of lung cancer patients and health controls detected by GC–MS. Scatterplots with boxplots showing the significantly changed metabolite classes in lung cancer. **B** Volcano plots of significantly up/down-regulated expressed metabolites in lung cancer. The most differentially expressed polar metabolites were mainly concentrated in 3 types of metabolites: carbohydrates, amino acids and carboxylic acids (log2FC > 0.5 or log2FC < -0.5 and *p* < 0.05, compared with HC). **C** D-lyxose (FC = 5.17, *p* < 0.01), L-sorboase (FC = 1.66, *p* < 0.01), galactinol (FC = 1.65, *p* < 0.01), urea (FC = 1.54, *p* < 0.01), D-allose (FC = 1.50, *p* < 0.01), and L-threonine (FC = 1.46, *p* < 0.01) were the top 6 significantly increased metabolites in patients with lung cancer. **D** Maltitol (FC = 0.37, *p* < 0.01), palatinitol (FC = 0.38, *p* < 0.01), eicosapentaenoic acid (FC = 0.44, *p* < 0.01), D-mannitol (FC = 0.55, *p* < 0.01), glycolic acid (FC = 0.59, *p* < 0.01), and thymol (FC = 0.60, *p* < 0.01) were the top 6 significantly decreased metabolites in patients with lung cancer. **E** OPLS-DA score plot showed the high separating capacity of polar metabolites. **F** The top 6 up-regulated polar metabolites in VIP chart (VIP > 1, FC > 1, and *p* < 0.05). The top 6 up-regulated polar metabolites. **G** The top 5 scored down-regulated polar metabolites in VIP chart (VIP > 1, FC < 1, and *p* < 0.05). **H** Enrichment pathway analysis to identify signaling pathways related to changed metabolites. The 3 most significantly activated signaling pathways were arginine biosynthesis, aminoacyl-tRNA biosynthesis, and galactose metabolism. The 3 most significantly inhibited signaling pathways were glyoxylate and dicarboxylate metabolism, alanine, aspartate and glutamate metabolism, and arginine and proline metabolism. *p < 0.05; ** p < 0.01; *** *p* < 0.001

The comparison of average concentration levels of metabolite species between lung cancer patients and HC revealed the detection of 63 polar metabolites, out of which 33 exhibited significant differential expression (14 were higher and 19 were lower, respectively, compared to HC, all *p* < 0.05), demonstrating significant differences in abundance between lung cancer and controls. The plasma levels of alcohols, amides, and peptide metabolites of lung cancer patients (*n* = 111) were significantly increased than that of HC (*n* = 111), whereas carboxylic acids, hydrocarbons, and fatty acids were decreased (Fig. 2 panels A and B). Of those significantly changed polar metabolites in patients with lung cancer, the top 6 increased metabolites (D-lyxose, L-sorboase, galactinol, urea, D-allose, and L-threonine) and top 6 decreased metabolites (maltitol, palatinitol, eicosapentaenoic acid, D-mannitol, glycolic acid, and thymol), compared to HC, were also identified (Fig. 2 panels C and D). To identify differential metabolite species, OPLS-DA models were established and employed. Six metabolites, such as D-lyxose, galactinol, urea, D-allose, D-glucose, and D-mannose, were upregulated (all VIP > 1, FC > 1, and *p* < 0.05), while 5 metabolites, namely lactic acid, glycolic acid, D-mannitol, palatinitol, and maltitol, were down-regulated (all VIP > 1, FC < 1, and *p* < 0.05), defined on the basis of VIP score of lung cancer (Fig. 2 panel E). Furthermore, those remarkably changed metabolites were mapped to KEGG pathways using Metabol Analyst's pathway enrichment tool. Six upregulated pathways, including Arginine biosynthesis, Aminoacyl-tRNA biosynthesis, Galactose metabolism, Glycine, serine and threonine metabolism, Nitrogen metabolism, and D-Glutamine and D-glutamate metabolism, and 8 down-regulated pathways, including Glyoxylate and dicarboxylate metabolism, Alanine, aspartate and glutamate metabolism, Arginine and proline metabolism, Arginine biosynthesis, Citrate cycle (TCA cycle), Pyruvate metabolism, Glycolysis/Gluconeogenesis and Glycine, and serine and threonine metabolism, were identified (Fig. 2 panel H).

### Lipidomic profile of lung cancer

**Fig. 3:**
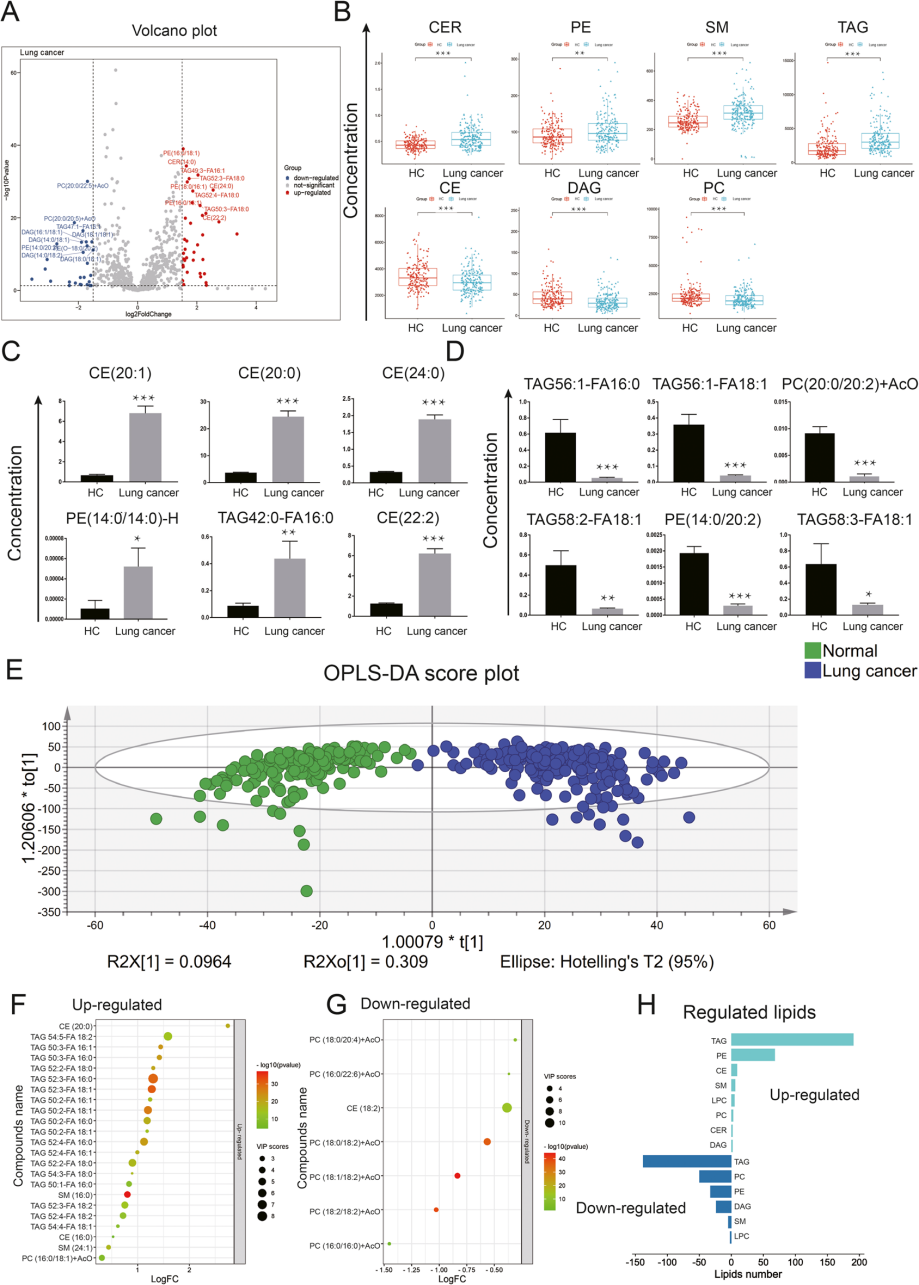
The lipidomics landscape of lung cancer. **A** Changes in lipid profiles in plasma of lung cancer patients and normal controls detected by LC–MS. Volcano plots of significantly up-/down-regulated expressed lipids in lung cancer group. TAGs were the most variable lipids, with multiple TAG contained in both elevated and reduced lipids (log2FC > 1.5 or log2FC < -1.5, and *p* < 0.05, compared with HC). **B** Scatterplots with boxplots showing the significantly changed lipid classes in lung cancer. **C** CE(20:1) (FC = 10.22, *p* < 0.01), CE(20:0) (FC = 6.70, *p* < 0.01), CE(24:0) (FC = 5.85, *p* < 0.01), PE(14:0/14:0)-H (FC = 5.00, *p* < 0.01), TAG42:0-FA16:0 (FC = 4.96, *p* < 0.01), and CE(22:2) (FC = 4.94, *p* < 0.01) were the top 6 significantly increased lipids in patients with lung cancer. **D** TAG56:1-FA16:0 (FC = 0.08, *p* < 0.01), TAG56:1-FA18:1 (FC = 0.12, *p* < 0.01), PC(20:0/20:2) + AcO (FC = 0.12, *p* < 0.01), TAG58:2-FA18:1 (FC = 0.13, *p* < 0.01), PE(14:0/20:2) (FC = 0.15, *p* < 0.01) and TAG58:3-FA18:1 (FC = 0.20, *p* < 0.01) were the top 6 significantly decreased lipids in patients with lung cancer. **E** The score plot generated by OPLS-DA demonstrated the strong discriminatory ability of lipids. **F** The top 23 up-regulated lipids in VIP chart (VIP > 1, FC > 1, and *p* < 0.05). **G** The top 7 down-regulated lipids in VIP chart (VIP > 1, FC < 1, and *p* < 0.05). **H** Among the up-/down-regulated lipids, the cumulative number of significantly changed lipids in each lipid class. The 3 lipid types that increased the most were TAG, PE and CE. The 3 most reduced lipid types were TAG, PC and PE. *p < 0.05; ** p < 0.01; *** *p* < 0.001

The intensity levels of CER, PE, SM, and TAG in the plasma of lung cancer patients (*n* = 204) were significantly higher than those of HC (*n* = 204), whereas the levels of CE, DAG and PC were remarkably reduced. Comparison of the mean concentration of lipid species between lung cancer patients and HC identified 742 lipids, of them 541 were differentially expressed (287 were higher and 254 were lower, respectively, compared to HC, all* p* < 0.05), indicating significant differences of abundance between lung cancer and HC (Fig. 3 panels A and B). Of these significantly changed lipids in patients with lung cancer, the top 6 elevated lipids and top 6 decreased lipids were also identified (Fig. 3, panels C and D): levels of CE(20:1), CE(20:0), CE(24:0), PE(14:0/14:0)-H, TAG42:0-FA16:0, and CE(22:2) were significantly increased, compared to that of HC (all* p* < 0.05); levels of TAG56:1-FA16:0, TAG56:1-FA18:1, PC(20:0/20:2) + AcO, TAG58:2-FA18:1, PE(14:0/20:2), and TAG58:3-FA18:1 were significantly decreased, comparing to that of HC (all* p* < 0.05). To better identify differential lipid species, OPLS-DA models were established and employed (Fig. 3, panel E). Twenty-three metabolites, including CE(20:0), TAG54:5-FA18:2, and TAG50:3-FA16:1, were upregulated (all VIP > 1, FC > 1, and *p* < 0.05); 7 metabolites, including PC(18:0/20:4), PC(16:0/22:6), and CE(18:2), were down-regulated (all VIP > 1, FC < 1, and *p* < 0.05), defined on the basis of VIP score of lung cancer (Fig. 3 panels F and G). Lastly, the numbers of lipids that were significantly up-or down-regulated in multiple lipid subclasses were counted (Fig. 3 panel H). Most of the significantly upregulated lipids exist in two major classes of lipids as TAG and PE, while the downregulated lipids mainly exist in TAG, PC, PE, and DAG.

### Correlation of polar metabolites and lipids

**Fig. 4:**
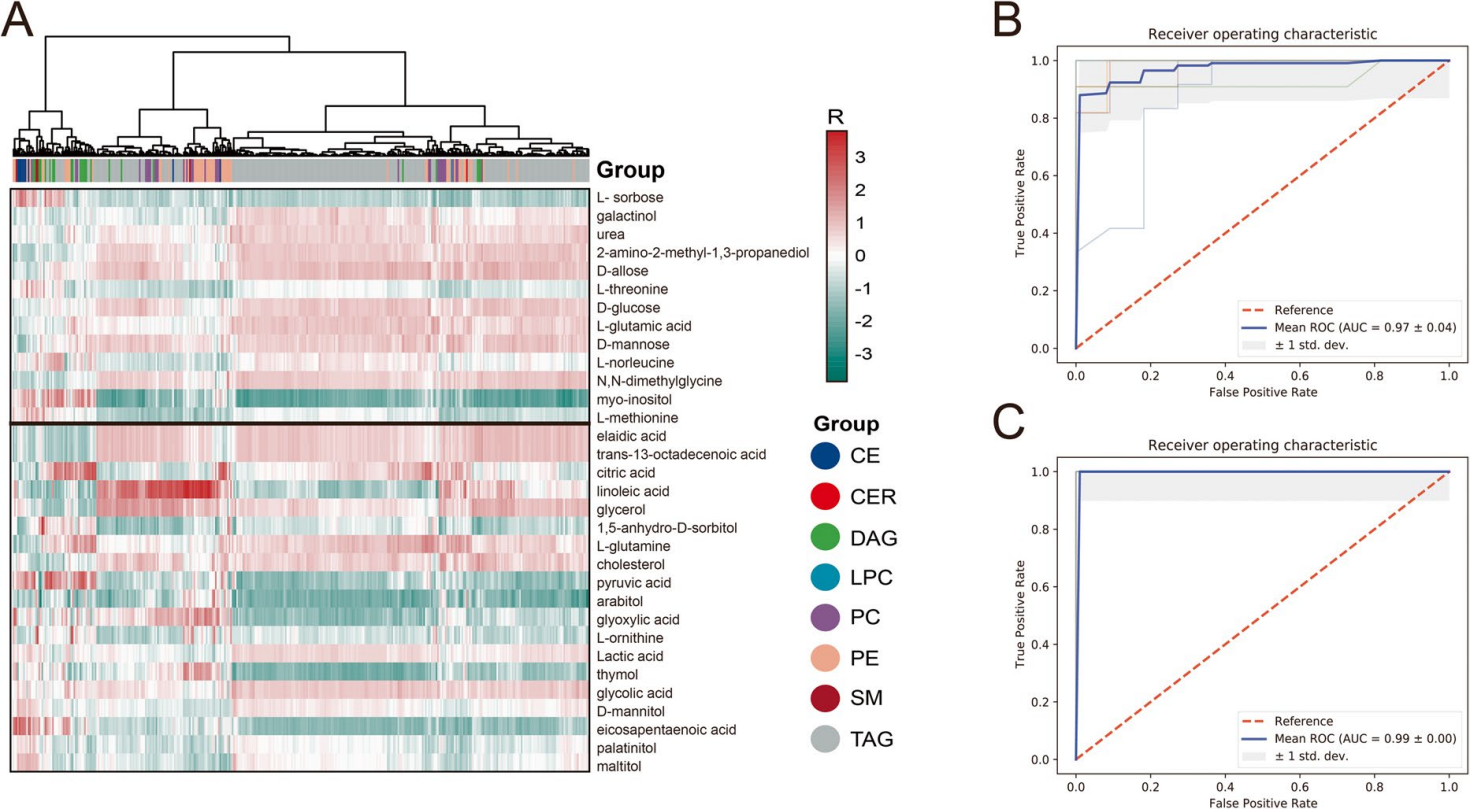
Correlation between polar metabolome and lipidome. Pearson correlation analysis examined the relationship between polar metabolomics and lipids. Heatmaps were used to show the relationship between these two omics results. The color gradients of the heatmap indicates the Pearson correlation coefficients. The horizontal line in the middle of heatmap is the dividing line between up and down regulated metabolites, and above the horizontal line is increased metabolites, below the horizontal line is decreased metabolites. A variety of polar metabolites were found to have a clear correlation with lipids (**A**). ROC curves of polar metabolites (**B**) and lipids (**C**) were constructed using tenfold cross-validated evaluation based on the random forests algorithm. The averaged ROC curves (represented in blue) were generated by calculating the mean of all ROC curves for polar metabolites or lipids. Our AUC was calculated using the mean ROC curve. AUC stands for the area under the curve, the shaded part is 1 standard deviation above and below. ROC curves demonstrated that the expression of polar metabolites and lipids could distinguish lung cancer patients from HC

Pearson correlation analysis examined the relationship between polar metabolomics (*p* < 0.05, compared to HC) and lipids (*p* < 0.05, logFC > 0.5 or logFC < -0.5, compared to HC), explored the complexity of metabolic networks in the plasma of lung cancer patients (Fig. 4 panel A), and revealed the significant correlations between a few metabolites and lipids. The top 5 significant lipids that were positively and negatively correlated with the expression of these up/down-regulated metabolites were listed in Table 1 with their functional associations and roles in cancers annotated. There was a significant difference in TAG levels between lung cancer and HC (*p* < 0.001). Up-regulated lipids with VIP > 1 and *p* < 0.05 were mainly concentrated in TAG. Although lipids in the lung cancer group generally had weak negative associations with the polar metabolites, a variety of metabolites were found strongly positively correlated with TAG. In lung cancer patients, the significant upregulated amino acids generally had a positive correlation with TAG44:0-FA16:0 and TAG48:2-FA18:0, and upregulated carbohydrates had a significant positive correlation with TAG51:1-FA18:0. A few carbohydrate metabolites that were significantly downregulated in the lung cancer group were positively associated with TAG44:1-FA14:0, TAG44:1-FA18:1, and TAG44:2-FA14:0. Down-regulated fatty acids that showed a general positive association with TAG44:1-FA14:0, TAG46:1-FA18:1, and TAG46:2-FA18:2.CE(22:0) was negatively correlated with the downregulated metabolite D-mannitol and pyruvic acid. The discriminative power of significant differenced metabolites (*p* < 0.05, Fig. 4, panel B) and lipids (*p* < 0.05, logFC > 0.5 or < -0.5, Fig. 4 panel C) were tested using mean ROC curve analysis generated by tenfold cross-validation, and found that both polar metabolites and lipids profiles could distinguish lung cancer from HC, in particular, lipids showed promising discriminative power with superior sensitivity and specificity.

**Table 1:** The top 5 significant lipids that were positively and negatively correlated with the expression of these up/down-regulated metabolites

Metabolite	Positive correlation	Negative correlation	Function
L- sorbose	CE(24:0), CE(22:2), DAG(20:0/20:0), CE(18:0)		
Galactinol	TAG44:1-FA18:1, TAG44:2-FA14:0, TAG48:2-FA18:0, TAG44:2-FA18:2, TAG46:2-FA18:1		Galactinol helps cancer cells maintain high glycolysis activity and promote cancer progression (Xu et al. 2020)
Urea	TAG44:1-FA18:1, TAG46:2-FA18:1, TAG48:2-FA18:0, TAG44:2-FA18:2, TAG44:2-FA14:0		Urea associated with mortality in patients with SCLC, and elevated plasma urea is a significant predictor of early mortality in patients (Winter et al. 2008)
2-amino-2-methyl-1,3-propanediol	TAG48:2-FA18:0, TAG44:1-FA14:0, TAG56:7-FA20:5, TAG46:2-FA18:2, TAG46:1-FA18:1		
D-allose	TAG51:1-FA18:0, TAG46:1-FA18:1, TAG44:1-FA14:0, TAG49:2-FA14:0, TAG50:3-FA18:0		D-allose have growth inhibitory effects in malignancies; D-allose inhibited NSCLC cell proliferation and tumour progression; In combination with cisplatin, D-allose had an additional antitumour effect (Kanaji et al. 2018)
L-threonine	TAG44:2-FA14:0, TAG44:1-FA14:0, PE(14:0/20:5), TAG47:1-FA14:0, TAG44:0-FA16:0		A modified bioisoster of L-threonine can selectively kill signet ring cancer cells without harming normal cells (Olgun 2019)
D-glucose	TAG46:1-FA18:1, TAG44:1-FA14:0, TAG51:1-FA18:0, TAG49:2-FA14:0, TAG49:2-FA18:2		Patients with lung cancer have increased glucose levels; fasting blood glucose can predict the survival of patients with locally advanced NSCLC undergoing concurrent chemoradiotherapy (Bergamino et al. 2019)
L-glutamic acid	TAG44:1-FA18:1, TAG53:1-FA18:0, TAG48:2-FA18:0, TAG46:2-FA18:1, TAG51:1-FA18:0		L-glutamic acid concentration distinguishes lung cancer from pneumonia and serves as a complementary tool to differentiate benign pet-positive lung lesions from lung cancer (Vanhove et al. 2018)
D-mannose	TAG48:2-FA18:0, TAG51:1-FA18:0, TAG44:1.FA18.1, TAG48:3-FA18:1, TAG44:2-FA14:0		D-mannose is a novel prognostic biomarker for patients with esophageal adenocarcinoma (Gu et al. 2017)
L-norleucine	TAG44:2-FA14:0, TAG44:0-FA16:0, TAG46:2-FA16:0, TAG53:1-FA18:0, TAG48:2-FA18:0		
N,N-dimethylglycine	TAG56:7-FA20:5, TAG48:2-FA18:0, TAG44:1-FA18:1, TAG46:2-FA18:2, TAG44.2.FA14.0		
Myo-inositol	DAG(20:0/20:0), TAG52:0-FA20:0, PC(20:0/20:3) + AcO, TAG48:2-FA14:0, TAG50:2-FA18:1	TAG54:7-FA20:5, TAG58:7-FA18:2, TAG56:6-FA18:0, TAG56:7-FA20:5, TAG56:8-FA20:5	Plasma inositol can be used as an indicator for the identification of prostate cancer and benign prostatic hyperplasia (Wang et al. 2021)
L-methionine	TAG44:2-FA14:0, TAG44:0-FA16:0, TAG44:1-FA18:1, TAG48:2-FA18:0, TAG46:1-FA18:1	PE(P + 16:0/16:1), TAG58:9-FA22:6, TAG58:9-FA18:2, TAG58:10-FA22:6, PE(P + 16:0/18:0)	L-methionine and its metabolites inhibits proliferation of breast, prostate, pancreatic and colon cancer cells (Lu et al. 2020)
Elaidic acid	TAG48:2-FA18:0, TAG46:2-FA18:2, TAG48:3-FA18:1, TAG46:1-FA18:1, TAG44:1-FA14:0		Elaidic acid promotes metastasis of colorectal cancer cells (Ohmori et al. 2017)
Trans-13-octadecenoic acid	TAG48:2-FA18:0, TAG46:2-FA18:2, TAG48:3-FA18:1, TAG46:1-FA18:1, TAG44:1-FA14:0		
Citric acid	TAG50:4-FA20:4, TAG49:0-FA16:0, TAG49:1-FA17:0, TAG52:4-FA20:4, TAG48:1-FA18:0		Changes in plasma citric acid levels in breast cancer patients are associated with anthracycline cardiotoxicity (Asnani et al. 2020)
Linoleic acid	PE(18:2/20:5), PC(20:0/18:1) + AcO, TAG54:5-FA18:2, PE(18:2/20:1), PC(20:0/22:4) + AcO		Linoleic acid induces migration and invasion in breast cancer cells (Rodriguez-Monterrosas et al. 2018)
Glycerol	TAG48:3-FA18:1, TAG48:2-FA18:0, TAG48:4-FA18:1, TAG46:2-FA18:2, TAG48:5-FA18:3		Oral administration may inhibit cancer progression (Capiglioni et al. 2018)
1,5-anhydro-D-sorbitol	TAG42:1-FA16:0, TAG44:2-FA16:0, TAG42:0-FA16:0	DAG(18:1/22:6), TAG58:10-FA18:2, CE(22:0), DAG(16:0/22:6), TAG58:10-FA22:6	
L-glutamine	TAG53:1-FA18:0, TAG51:1-FA18:0, TAG44:1-FA18:1, TAG48:1-FA18:0, TAG50:4-FA20:4		Cancer cells use glutamine to meet their metabolic needs (Marin-Aguilera et al. 2021)
Cholesterol	TAG44:1-FA14:0, TAG46:1-FA18:1, TAG56:7-FA20:5, TAG48:2-FA18:0, TAG46:2-FA18:2		Cholesterol-derived metabolites play complex roles in supporting cancer progression and suppressing immune responses; Cholesterol promotes tumor formation and growth (Huang et al. 2020)
Pyruvic acid	DAG(20:0/20:0), TAG50:0-FA14:0, CE(18:0), PC(20:0/20:3) + AcO, DAG(16:0/22:5)	PE(O + 18:0/16:1), TAG56:7-FA18:0	Pyruvic acid has been demonstrated to be an important cancer biomarker (Alizadeh and Nayeri 2020)
Arabitol		DAG(16:1/18:2), TAG56:9-FA18″3, CE(22:0), TAG52:8-FA16:1, PE(O + 16:0/18:3)	Decreased arabitol levels in patients with pathological complete remission after adjuvant chemoradiotherapy (Fujigaki et al. 2018)
Glyoxylic acid	DAG(18:2/22:4)		
L-ornithine	SM(20:0), TAG44:2-FA14:0, TAG48:2-FA18:0, TAG46:2-FA16:0, DAG(18:2–18:3)		Increased l-ornithine affects the vasculature, causes neuronal toxicity, and causes abnormal growth of tumor cells (Caldwell et al. 2018)
Lactic acid	TAG44:1-FA18:1, TAG44:2-FA14:0, TAG46:2-FA18:1, TAG44:2-FA18:2, TAG44:1-FA14:0		Hyperlactatemia is an independent predictor of poor survival in metastatic lung cancer patients; Lactic acid promotes cancer progression (Certo et al. 2021)
Thymol	DAG(18:1/20:3)		Thymol induces mitochondrial pathway-mediated apoptosis in lung cancer cells (Balan et al. 2021)
Glycolic acid	TAG44:1-FA18:1, TAG51:1-FA18:0, TAG46:1-FA18:1, TAG46:2-FA18:1, TAG44:2-FA14:0		
D-mannitol	TAG44:2-FA14:0, TAG44:1-FA18:1, TAG44:1-FA14:0, TAG46:1-FA18:1, TAG51:1-FA18:0		
Eicosapentaenoic acid	CE(20:0), CE(22:2), CE(24:0), SM(26:0), CE(18:0)	PC(18:0/16:1) + AcO	Eicosapentaenoic acid inhibits tumor growth (Yang et al. 2014)
Palatinitol	TAG44:2-FA14:0, TAG44:1-FA14:0, TAG56:7-FA20:5, TAG44:1-FA18:1, TAG51:1-FA18:0		
Maltitol	TAG44:2-FA14:0, TAG44:1-FA18:1, TAG44:1-FA14:0, TAG48:2-FA18:0, TAG46:1-FA18:1		

### Trans-omic profiles integrating lipidomes with clinical phenomes

**Fig. 5:**
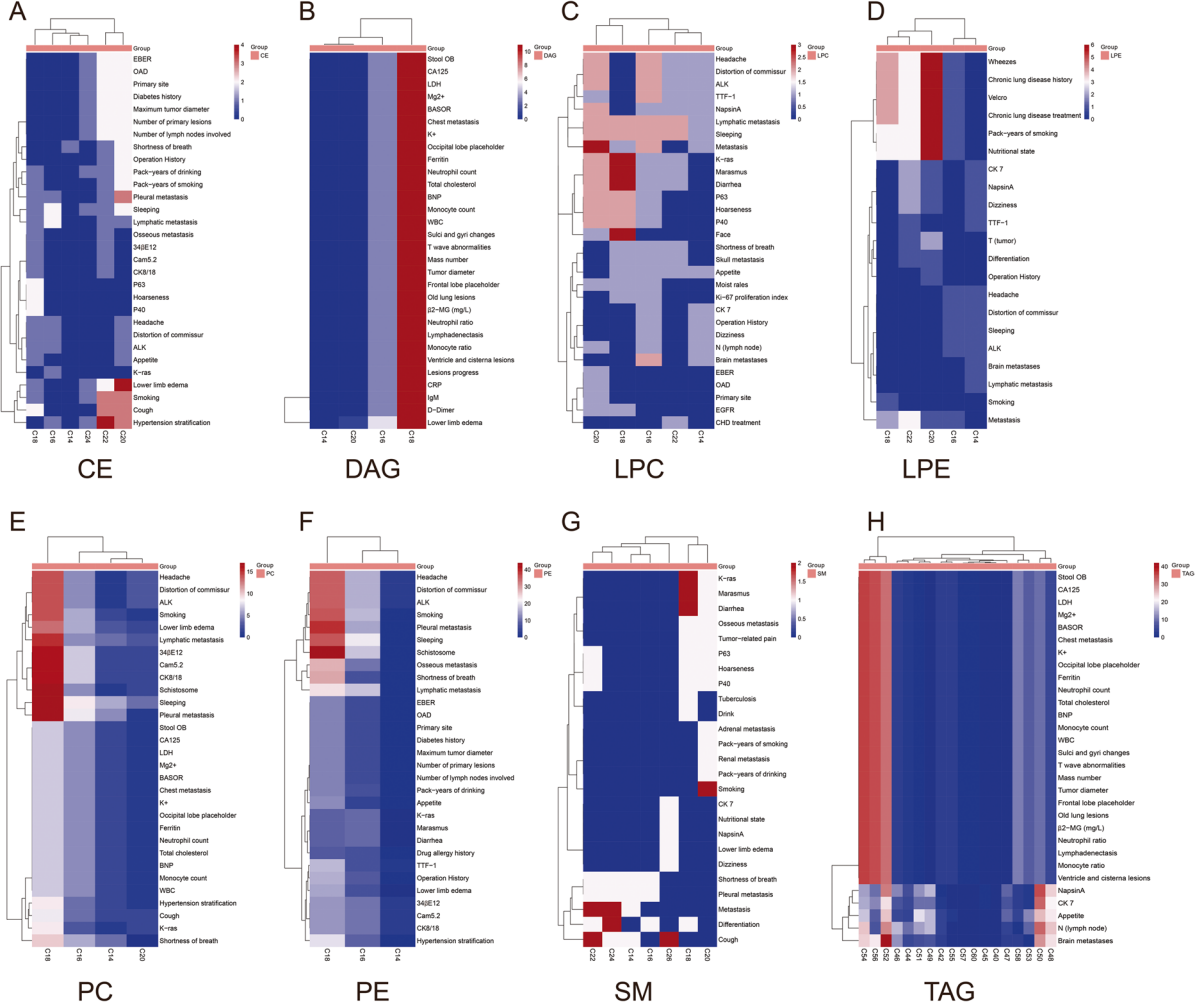
Trans-omic nodules cross-clinical phenomes and each lipid class measured by simulating the expression quantitative trait locus (eQTL) model. Heatmap showing the associations between the clinical phenomes and CE (**A**), DAG (**B**), LPC (**C**), LPE (**D**), PC (**E**), PE (**F**), SM (**G**) and TAG (**H**). The bluer the color of the heatmap, the weaker the correlation between the clinical phenomes and lipids; and the redder the color of the heatmap, the stronger the correlation between clinical phenomes and lipids

Based on the eQTL model, we modified the lipid quantitative trait loci model to investigate lipid-clinical phenomes correlation. In addition, MatrixlQTL R package was used to obtain the significant phenome-lipid pairs and their *p* values. Results showed that CE(C = 22, 24) were significantly associated with lower limb edema, smoking, cough, and hypertension stratification (Fig. 5, panel A). The close correlation between DAG and clinical phenomes focused on C18 (Fig. 5, panel B). Metastasis was related to the level of LPC(C = 20) (Fig. 5, panel C). Marasmus and diarrhea were associated with LPC(C = 18) and SM(C = 18) (Fig. 5, panels C and G). Pulmonary-related phenotypes including wheeze, chronic lung disease history, Velcro, pack-years of smoking, and nutritional state were significantly associated with LPE(C = 20) (Fig. 5 panel D). Pathological examination results, ALK, 34βE12, Cam5.2, CK8/18, and lymphatic pleural metastasis were related to PC(C = 18) (Fig. 5 panel E). PE(C = 18) was associated with a variety of clinical phenotypes, including schistosome and pleural metastasis (Fig. 5, panel F). TAG(C = 52, 54, and 56) mainly affect clinical phenotype (Fig. 5, panel H).

**Fig. 6:**
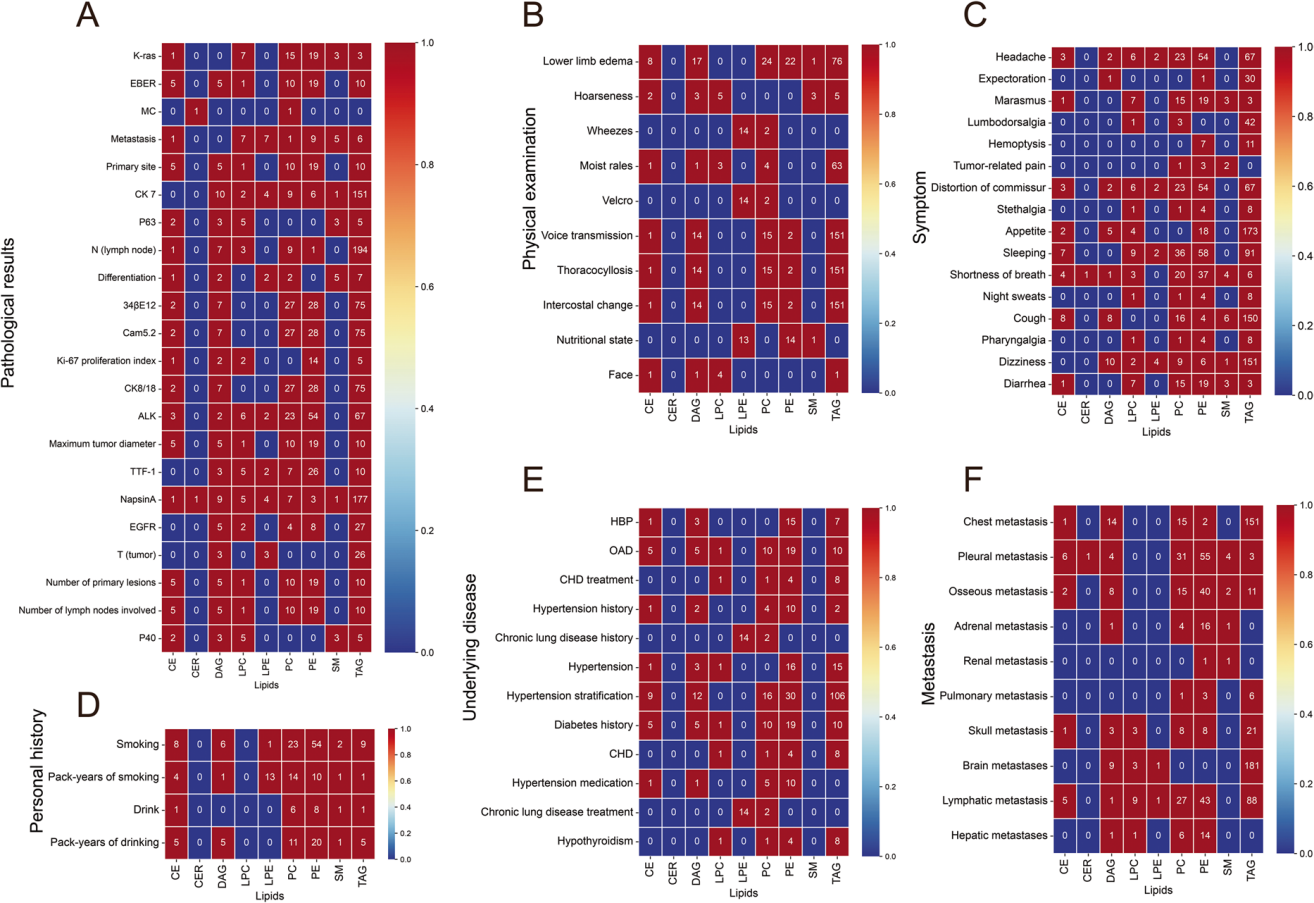
Correlations between various clinical phenotypes and lipids. Heatmap showing the associations between lipid classes and pathological results (**A**), physical examination (**B**), symptom (**C**), personal history (**D**), underlying disease (**E**), and metastasis (**F**). Numbers in the heatmap represent the number of related lipids detected per lipid class. The bluer the color of the heatmap, the less lipids are associated with the clinical phenotypes; and the redder the color of the heatmap, the more lipids are associated with the clinical phenotypes

The phenotype-related lipid classes were classified by different clinical phenotypes. TAG was found to be the most abundant lipid class in plasma, and most of the lipids that associated with pathological results, physical examination, symptoms, primary disease, and metastasis belonged to TAG. The results revealed that patients with positive expression of Napsin A had a strong correlation with lipids (Fig. 6, panel A). The lipids most relevant to physical examination were DAG, PC, and TAG. Among these phenotypes, lower limb edema, voice transmission, thoracocyllosis, and intercostal changes were associated with lipids (Fig. 6, panel B). Most of the symptoms associated with lung cancer were significantly related to PE in addition to TAG (Fig. 6, panel C). In patients who had a history of drinking or smoking, PC and PE were dysfunctional (Fig. 6, panel D). In patients with underlying disease, PE was prone to be abnormal (Fig. 6, panel E). PC, PE, and TAG were associated with tumor metastasis, especially pleural and osseous metastasis. Lung cancer was frequently accompanied by these two types of metastasis (Fig. 6, panel F).

### Lipidomic profiles of lung cancer subtypes

**Fig. 7:**
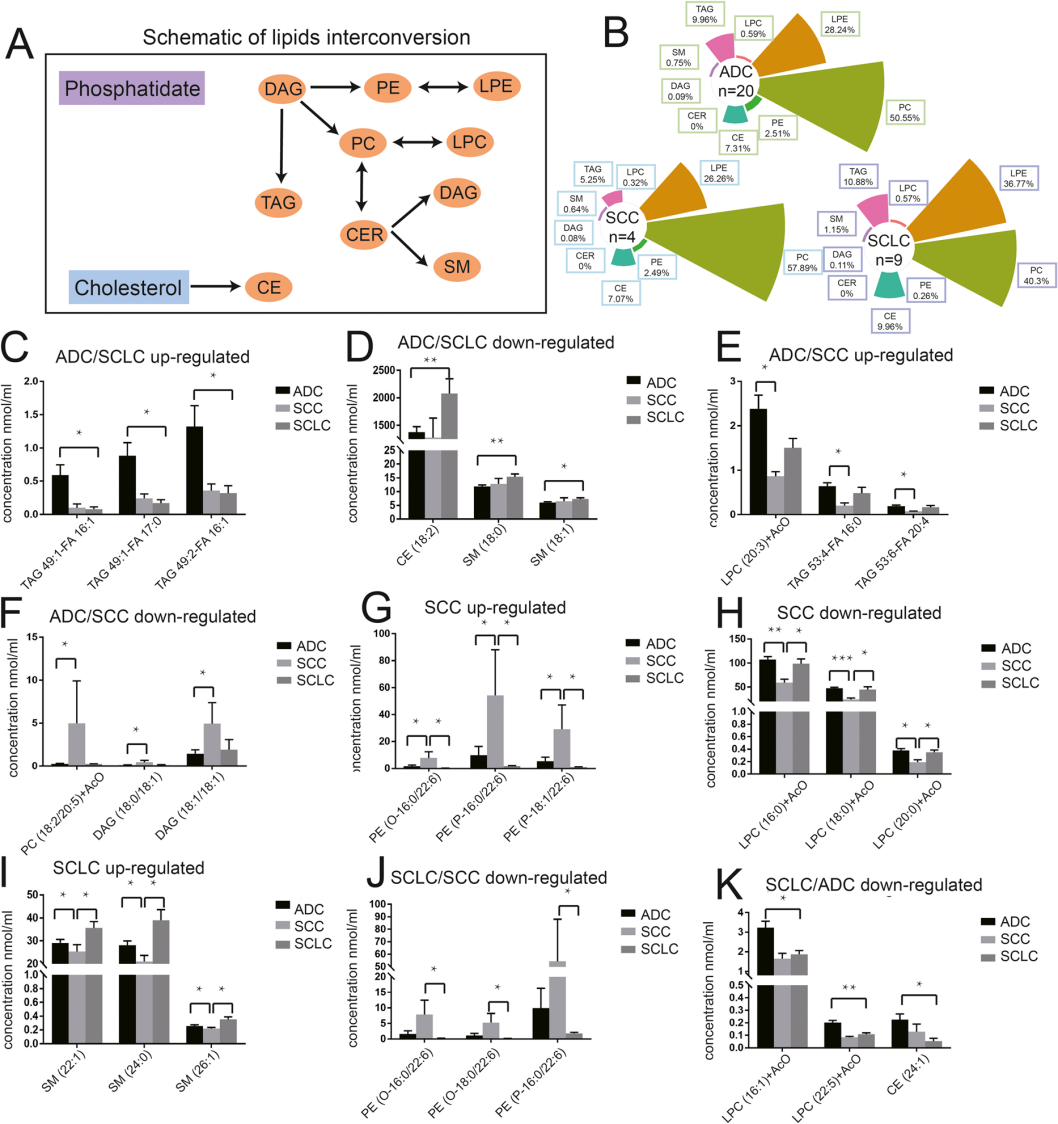
Top 3 significantly changed lipids in patients with different lung cancer subtypes. Schematic of interconversions among different lipids. DAG is derived from phosphatidate and converts into PE, PC and TAG. PC and PE further converts into LPC and LPE, respectively. In addition, PC converts into CER to generate DAG and SM. And cholesterol produces CE (**A**). Pie charts showing the proportions of multiple lipid classes in patients with different lung cancer subtypes (**B**). TAG49:1-FA16:1, TAG49:1-FA17:0, and TAG49:2-FA16:1 were the top 3 up-regulated lipids in ADC compared to SCLC (**C**). CE(18:2), SM(18:0), and SM(18:1) were the top 3 down-regulated lipids in ADC compared to SCLC (**D**). LPC(20:3), TAG53:4-FA16:0 and TAG53:6-FA20:4 were the top 3 up-regulated lipids in ADC compared to SCC (**E**). PC(18:2/20:5), DAG(18:0/18:1), and DAG(18:1/18:1) were the top 3 down-regulated lipids in ADC compared to SCC (**F**). PE(O-16:0/22:6), PE(P-16:0/22:6), and PE(P-18:1/22:6) were the top 3 up-regulated lipids in SCC compared to ADC and SCLC (**G**). LPC(16:0), LPC(18:0) and LPC(20:0) were the top 3 down-regulated lipids in SCC compared to ADC and SCLC (**H**). SM(22:1), SM(24:0), and SM(26:1) were the top 3 up-regulated lipids n SCLC compared to ADC and SCC (**I**). PE(O-16:0/22:6), PE(O-18:0/22:6), and PE(P-16:0/22:6) were the top 3 down-regulated lipids in SCLC compared to SCC (**J**). LPC(16:1), LPC(22:5), and CE(24:1) were the top 3 down-regulated lipids n SCLC compared to ADC (K). For all comparisons, *p < 0.05,**p < 0.01,***p < 0.001

Different lipids are generally interconverted by specific cellular lipid phosphatases and kinases. DAG is derived from phosphatidate and converts into PE, PC, and TAG. PC and PE further convert into LPC and LPE, respectively. In addition, PC converts into CER to generate DAG and SM. And cholesterol produces CE (Fig. 7, panel A). The proportions (%) of the 9 main lipid elements of ADC (*n* = 20), SCC (*n* = 4), and SCLC (*n* = 9) were plotted through the pie charts (Fig. 7 panel B). Notably, SM, CE and LPE increased mainly in SCLC, whereas PC and PE significantly decreased, compared with ADC and SCC. In addition, LPC and TAG were decreased in SCC. Differences exist in the changed lipids among lung cancer subtypes. Among the lipid elements that showed significant changes in lung cancer, the top three lipids from each group were identified. Levels of TAG(49:1-FA16:1, 49:1-FA17:0, and 49:2-FA16:1) were significantly higher (Fig. 7, panel C), and TAG(53:4-FA16:0, 53:6-FA20:4), LPC(20:3) were lower (Fig. 7 panel D) in ADC as compared with that of SCLC. Levels of CE(18:2), SM(18:0, and 18:1) were higher (Fig. 7, panel E), and PC(18:2/20:5) and DAG(18:0/18:1, and 18:1/18:1) were lower (Fig. 7 panel F) in ADC as compared with that of SCC. Levels of PE(O-16:0/22:6, P-16:0/22:6, and P-18:1/22:6) in SCC were up-regulated (Fig. 7 panel G), and levels of LPC(16:0, 18:0, and 20:0) in SCC were down-regulated (Fig. 7 panel H), when compared with that of ADC and SCLC. In SCLC, the levels of SM(22:1, 24:0, and 26:1) were increased compared to the groups of ADC and SCC (Fig. 7, panel I). Moreover, the levels of PE(O-16:0/22:6, O-18:0/22:6, and P-16:0/22:6) were decreased in SCLC when compared with SCC (Fig. 7 panel J). Levels of LPC(16:1 and 22:5), and CE(24:1) were also decreased when compared with ADC (Fig. 7, panel K).

## Discussion

Metabolic disturbance has shown potential for cancer diagnosis. Given its importance, studies to define the role of metabolic disturbances in lung cancer are warranted, yet related literatures have been limited. In the present study, a comprehensive metabolomic network was constructed to systematically describe the metabolomic landscape of lung cancer. We also investigated the association between polar metabolites and lipids, effects of lipids on clinical phenotypes, and differences in lipidomic profiles between subtypes of lung cancer. The study revealed that the changes in clinical phenotypes may be the result of the combined effects of metabolomics and lipidomics, clinical phenotypes including pathological results, physical examination, symptoms, primary disease and metastasis were significantly associated with TAG(especially with carbon number 52, 54, and 56), and TAG expression were strongly positively correlated with multiple polar metabolites. Overall, our study illustrated the metabolomic landscape of lung cancer, highlighted the potential application of plasma metabolites, including polar metabolites and lipids, to differentiate and diagnose lung cancer, and paved the way for developing a novel targeted therapies of lung cancer.

The discovery of plasma biomarkers has been heralding the ability to differentiate lung cancer patients from healthy or non-malignant lung disease patients by metabolomics and lipidomics for last decade (Zhang et al. 2020). In patients with non-squamous non-small cell lung cancer, a majority of functions related to carbohydrate, amino acid, and nucleotide pathways demonstrated an association with shorter overall survival (OS) (Ivanina Foureau et al. 2022). Increasing evidence found the alterations in plasma lipid metabolism in lung cancer patients (Lv et al. 2018a, b; Yu et al. 2024; Zhu et al. 2020). Due to the metabolic heterogeneity of lung cancer, the sensitivity, specificity, and clinical efficacy of a single marker is relatively limited. The combined applications of multiple metabolic biomarkers could enhance the precision and sensitivity of early screening while contributing to the advancement of personalized treatment approaches. Systematic studies have been undertaken to explore the potential significance of serum metabolites and lipids in the diagnosis of lung cancer. Among these studies, eight specific metabolites have been identified, including 1-mristoyl-sn-glycero-3-phosphocholine, 16b-hydroxyestradiol, 3-phosphoserine, cholesteryl sulfate, D-lyxose, dioctyl phthalate, DL-lactate and Leu-Phe were identifed for their potential value in the early diagnosis of SCLC (Shang et al. 2023). LPC 18:0, L-Phenylalanine, oxaloacetic acid and xanthine were also found to be significantly altered in the plasma of patients with non-small cell lung cancer (NSCLC) (Cang et al. 2022). However, to date, there are no studies that have combined metabolomics, lipidomics and clinical phenotypes in lung cancer patients. By integrating metabolomics and lipidomics data with clinical phenotype of patients, the present study constructed a comprehensive functional metabolic network of lung cancer. Analyzing metabolic biomarkers in conjunction with clinical phenotypes can help to better understand the mechanisms of lung cancer, its diagnosis, progression prediction, and therapeutic response, and facilitate the application of biomarkers in clinical practice.

In the current study, 63 polar metabolites and 742 lipids in the plasma of lung cancer patients and HC were quantified using LC–MS and GC–MS. Multi-omics data analysis provided a clear differentiation of the two groups. The polar metabolic profiling found that the expression of alcohols, amides, and peptides were increased, whereas carboxylic acids, hydrocarbons and fatty acids were decreased in lung cancer patients, compared to that of HC. In addition, plasma levels of various amino acids such as L-threonine and L-glutamic acid of lung cancer patients were increased compared to HC. Previous studies have reported that L-glutamic acid exhibits the potential to differentiate lung cancer from pneumonia. Consequently, it could serve as a supplementary tool to distinguish benign PET-positive lung lesions from lung cancer (Vanhove et al. 2018). Although the main energy supply of cancer cells is considered from glucose decomposing, recent studies found that amino acids are the largest source of nutrients for cancer cells. Furthermore, pathway enrichment analysis revealed that arginine biosynthesis, glyoxylate and dicarboxylate metabolism were dysregulated in lung cancer. In cancer cells, arginine and its metabolites regulate proliferation, growth, autophagy, apoptosis, and metastasis (Yang et al. 2020a, b). Arginine biosynthesis pathway is also associated with resistance to chemotherapy (Liu et al. 2021a, b). Glyoxylate and dicarboxylate metabolism disorders would decrease the ability to detoxify reactive oxygen species generated by chemotherapy and radiotherapy, leading to cancer-causing mutations (Cano et al. 2011). Taken together, the current study documented the disturbance of polar metabolites in lung cancer and identified L-threonine and L-glutamic acid as potential markers for lung cancer diagnosis.

Current lipidomics studies revealed remarkable differences that mainly concentrated in glycerolipid metabolism and glycerophospholipid metabolism between lung cancer patients and HC. Lipids are made up of fatty acids and other compounds. Fatty acids are the main components of lipids and they are organic acids consisting of carbon chains and carboxylic acid groups. The characteristics of fatty acids depend on their chain length, bond length, and saturation. Changes in these characteristics can modulate the structure and properties of lipids, thereby affecting their function and effects in organisms (Wenk 2005). A widest amplitude of TAG changes was found in lung cancer. TAGs were the most significantly changed lipid classes, both in up- and down-regulated lipids. Additionally, TAGs concentrations displayed significant relationship with the severity of clinical signs. TAGs are the primary storage form of intracellular lipids, and its species concentration varies considerably. The abnormal concentration of TAG is related to the morbidity and mortality of tumors and is considered an ideal target for cancer immunotherapy. PC and PE are the most abundant phospholipids and exhibit marked changes during the progression of many diseases. With oncogenesis and tumor progression, the rate of phospholipid synthesis increases. Increased concentrations of PE and PC have been detected in numerous cancer types. The dysregulation of PE and PC metabolism can be understood as a necessity to accommodate the rapid proliferation rate of cancer cells. Changes in chain length can affect multiple aspects of lipids, including physical properties, solubility, bioavailability and metabolism, and bioactivity (Wang et al. 2024). In present study, PEs were found significantly upregulated in lung cancer, but multiple PCs were downregulated, especially C16-C18 PC, including PC(18:0/20:4, 16:0/22:6, 18:0/18:2, 18:1/18:2, 18:2/18:2 and 16:0/16:0). This may be due to the reduction of PC after anticancer treatment (Morse et al. 2007). Fatty acids with carbon number between 16 and 18 are medium-length fatty acids that are relatively abundant in many foods and have relatively high stability. They provide the energy needed by the body and are essential for cell structure and function. Some specific fatty acids with carbon atoms between 16 and 18, such as linoleic acid, are thought to have immunomodulatory and anti-inflammatory effects (Villacorta et al. 2018). In line with our observations, Chen et al. reported a notable elevation in the levels of PEs among early-stage lung cancer patients (Chen et al. 2018). In addition, CEs (specially 20:0) were found to be significantly increased in lung cancer, suggesting that the accumulation of CE is a common indicator of cancer (Huang et al. 2020). The interrelations among different lipid classes were complex, full understanding of the lipid alterations in lung cancer requires further study.

Lipids can be converted from carbohydrates or amino acid metabolites. To determine the links between metabolomics and lipidomics, a correlation analysis was carried out to identify carbohydrates showing significant activation in lung cancer. Results found that most of the carbohydrates were positively correlated with TAGs. Increased carbohydrate intake and metabolism have been reported to be prominent metabolic features of most cancers. Previous research has established that increased levels of plasma carbohydrates play a role in the proliferation and migration of lung cancer cells. In the current study, a positive correlation was identified between the expression of several amino acids (such as L-threonine, L-glutamic acid, L-methionine, L-glutamine, and L-ornithine) and TAG levels. Amino acids, especially L-glutamic acid, not only provide energy, but also involve in the processes of protein folding, de novo nucleic acid synthesis, and lipid synthesis. Changes in amino acid content can regulate lipid synthesis. As there is no literature that has studied the association between TAG and glucose, the current analysis of the associations between metabolites and lipids would provide important clues to investigate the driving force of metabolic reprogramming in lung cancer.

To integrate lipidomics with clinical information, the study scored clinical phenomes by DESS to combine lipidomic data with clinical phenomes of lung cancer patients. Using the modified eQTL model, and MatrixEQTL as an achievable additive linear model (Zhang et al. 2020; Zhu et al. 2020), correlations between clinical phenomena and lipid molecules were calculated, and patient phenome-specific lipid elements were subsequently identified. As one clinical phenotype may be associated with changes in multiple lipid components, and changes in one lipid component may affect multiple clinical phenotypes, alterations of lipids may be disease-specific or phenomenon-specific, thus their validity as diagnostic biomarkers requires further investigation. The number of specific lipids found corresponding to the clinical phenomena in the study testified the complexity of the regulatory mechanisms. The study found that headache was corresponded with PC and PE, and headache-specific lipid elements profiles were further examined. Classified by carbon chain length, PC(C = 18) and PE(C = 18) were found to be the lipids most frequently associated with headache. These results suggested that different metabolic pathways and metabolites contributed to the same clinical phenotype. Conversely, the same metabolic pathways and metabolites can manifest in different clinical phenomena. For example, LPE(C = 20) was found to be involved in the appearance of wheeze, chronic lung disease, velcro, smoking, and nutritional state. Cancer cells exhibit alterations in their metabolism in response to both internal and external microenvironments, which are regulated by the modulation of the trans-omic network. In order to achieve a comprehensive comprehension of the involved biological systems, it is imperative to perform an extensive analysis of pertinent omics datasets. The integration of multiple omics data, known as trans-omics, provides a synergistic effect that can contribute to the development of precise medicine for lung cancer (Zhang et al. 2020). Cancer cells enhance lipogenesis to support their rapid proliferation. The expressions of corresponding genes and epigenetic modifications can regulate lipid metabolites and the associated restriction enzymes. The current study demonstrated the manifold interactions between developing tumors and the metabolic microenvironment and supported the significance of combining multiple metabolomics with clinical phenoms in understanding the molecular mechanisms of lung cancer pathogenesis.

We also compared lipid profiles in different lung cancer subtypes. Results showed that LPC(16:0, 18:0, 20:0) were remarkably downregulated in SCC. Decreased LPCs were often found in patients with early stage lung cancer (Ros-Mazurczyk et al. 2017). Moreover, a large-scale study suggested that a lower level of plasma LPCs, especially LPC(18:0), could be associated with increased risk of breast, prostate, and colorectal cancer. In contrast, higher levels of certain PC were associated with increased cancer risk (Kuhn et al. 2016). According to the annotation of registered masses in the human plasma lipidome database, several LPCs were down-regulated in cancers (Lv et al. 2018a, b). Compared with ADC and SCLC, SCC had significantly higher levels of PEs, mainly in PE(16:0/22:6) and PE(18:0/22:6). Although previous research found that PE(16:0/22:6, 18:0/22:6) were markedly increased in type 2 diabetes mellitus, this effect has not been reported in any lung cancer studies (Zhang et al. 2022). The present study revealed that epidemic profiling has a unique value for lung cancer early diagnostics, however its ununiformity needs to be cautiously considered, as this malignance is a highly heterogeneous disease.

The study should be viewed and interpreted in the light of its limitations. As the study was based on multiple metabolomicss integrated with clinical phenoms, such that the findings need to be further validated. There were compounding factors in the process of clinical phenomena and lipid profile integration, including limited sample size, especially for SCC and SCLC lung cancer patients, relatively low specificity of metabolites, and the variability of the disease itself. In addition, most of the enrolled lung cancer patients received chemotherapy treatment, which might have compromised the results. Despite these limitations, the present study, however, underscores the potential application of plasma metabolites, including polar metabolites and lipids, to differentiate and diagnose lung cancer. Furthermore, this study highlights the importance of utilizing trans-omics profiles that integrate clinical observations with lipidomics. This approach allows for the exploration of the heterogeneity in lipid metabolism within lung cancer and the identification of lipidome landmarks associated with specific clinical phenomena.

In summary, using a comprehensive multi-omics metabolomic dataset, the present study illustrated the plasma metabolomic landscape of lung cancer. By combining multi-omics profiling, the study also discovered potential metabolic diagnostic and therapeutic targets for lung cancer. Furthermore, the correlation of lipidomics with metabolomics and clinical phenotypes of the study would expand the knowledge pool of genome-based precision medicine for lung cancer. Future large sample size study is warranted due to the heterogeneous nature of this malignancy.

## Supplementary Information

Below is the link to the electronic supplementary material.

Supplementary file1 (DOCX 33 KB)

## Data Availability

The datasets used and/or analyzed during the current study are available from the corresponding author on reasonable request.

## Abbreviations

*PC*: Phosphatidylcholines
*PE*: Phosphatidylethanolamine
*LPC*: Lysophosphatidylcholine
*LPE*: Lysophosphatidylethanolamine
*SM*: Sphingomyelin
*TAG*: Triacylglycerol
*DAG*: Diacylglycerol
*CE*: Cholesteryl ester
*ADC*: Lung adenocarcinoma
*SCC*: Squamous cell carcinomas
*SCLC*: Small cell lung cancer
*HC*: Healthy control
*DESS*: Digital Evaluation Score System
*GC-MS*: Gas chromatography mass spectrometry
*MSD*: Mass selective detector
*LC-MS*: Liquid chromatography mass spectrometry
*PCA*: Principal component analysis
*OPLS-DA*: Orthogonal projections to latent structures discriminate analysis
*VIP*: Variable importance projection
*FC*: Fold change
*eQTL*: Expression quantitative trait locus model
