# Supplementary material for: Integration of clinical phenoms and metabolomics facilitates precision medicine for lung cancer
Source: Cell Biol Toxicol. 2024 May 1;40(1):25. doi: 10.1007/s10565-024-09861-w (PMC11063108; doi:10.1007/s10565-024-09861-w)
Supplement: Supplementary file 1 — Supplementary file1 (DOCX 33 KB) [file 10565_2024_9861_MOESM1_ESM.docx]

**Supplementary Table 1. Clinical phenomes scored and collected in lung cancer patients**

**.**

| Clinical phenomes | DESS Scores | | | |
| --- | --- | --- | --- | --- |
|  | 0 | 1 | 2 | 4 |
| Basic information | | | | |
| Age | <40 | 40-49 | 50-74 | ≥75 |
| Length of stay | <1 | 1-3 | 4-7 | >7 |
| Symptoms | | | | |
| Shiver | no | slight | medium | severe |
| Dizziness | no | slight | medium | severe |
| Headache | no | slight | medium | severe |
| Hyposarca | no | slight | medium | severe |
| Tired | no | slight | medium | severe |
| Fever | no | slight | medium | severe |
| Disturbance of consciousness | no | slight | medium | severe |
| Blurred vision | no | slight | medium | severe |
| Eye drying | no | slight | medium | severe |
| Tinnitus | no | slight | medium | severe |
| Amnesia | no | slight | medium | severe |
| Testiness | no | slight | medium | severe |
| Limitation of mouth opening | no | slight | medium | severe |
| Thirst | no | slight | medium | severe |
| Ozostomia | no | slight | medium | severe |
| Bitter taste | no | slight | medium | severe |
| Distortion of commissur | no | slight | medium | severe |
| Pharyngalgia | no | slight | medium | severe |
| Dysphagia | no | slight | medium | severe |
| Palpitation | no | slight | medium | severe |
| Stethalgia | no | slight | medium | severe |
| Chest distress | no | slight | medium | severe |
| Cough | no | slight | medium | severe |
| Expectoration | no | slight | medium | severe |
| Hemoptysis | no | slight | medium | severe |
| Shortness of breath | no | slight | medium | severe |
| Asthma | no | slight | medium | severe |
| Breathing difficulties | no | slight | medium | severe |
| Nausea | no | slight | medium | severe |
| Emesis | no | slight | medium | severe |
| Hemoptysis | no | slight | medium | severe |
| Acid reflux | no | slight | medium | severe |
| Bad appetite | no | slight | medium | severe |
| Abdominal distension | no | slight | medium | severe |
| Diarrhea | no | slight | medium | severe |
| Constipation | no | slight | medium | severe |
| Hematochezia | no | slight | medium | severe |
| Lumbodorsalgia | no | slight | medium | severe |
| Joint pain | no | slight | medium | severe |
| Frequency of urinatior | no | slight | medium | severe |
| Nocturia increased | no | slight | medium | severe |
| Odynuria | no | slight | medium | severe |
| Vesical tenesmus | no | slight | medium | severe |
| Hematuresis | no | slight | medium | severe |
| Uroclepsia | no | slight | medium | severe |
| Dysuresia | no | slight | medium | severe |
| Anemia | no | slight | medium | severe |
| Tumor-related pain | no | slight | medium | severe |
| Morning stiffness | no | slight | medium | severe |
| Joint swelling | no | slight | medium | severe |
| Limbs weakness | no | slight | medium | severe |
| Intermittent claudication | no | slight | medium | severe |
| Rest pain | no | slight | medium | severe |
| Limbs numbness | no | slight | medium | severe |
| Limbs pain | no | slight | medium | severe |
| Limbs cold | no | slight | medium | severe |
| Muscle spasm | no | slight | medium | severe |
| Muscle tremors | no | slight | medium | severe |
| Sleeping | no | slight | medium | severe |
| Night sweat | no | slight | medium | severe |
| Marasmus | no | slight | medium | severe |
| Obesity | no | slight | medium | severe |
| Tetter | no | slight | medium | severe |
| Ecchymosis | no | slight | medium | severe |
| Underlying diseases | | | | |
| Hypertension | no | ≤5 years | 5-10 years | ≥10 years |
| Diabetes | no | ≤5 years | 5-10 years | ≥10 years |
| High blood pressure | no | Level 1 | Level 2 | Level 3 |
| Hypertension medication | no | 1 treatment | 2 treatments | 3 or more treatments |
| Hypertension stratification | low-risk | slight-risk | medium-risk | severe-risk |
| Oral hypoglycemic drug | no | 1 treatment | 2 treatments | 3 or more treatments |
| hyperlipidaemia | no | ≤5 years | 5-10 years | ≥10 years |
| hyperuricemia | no | ≤5 years | 5-10 years | ≥10 years |
| Cerebral infarction | no | ≤5 years | 5-10 years | ≥10 years |
| Chronic lung disease | no | ≤5 years | 5-10 years | ≥10 years |
| Hypothyroidism | no | ≤5 years | 5-10 years | ≥10 years |
| Hyperthyroidism | no | ≤5 years | 5-10 years | ≥10 years |
| Nephropathy | no | ≤5 years | 5-10 years | ≥10 years |
| Autoimmune disease | no | ≤5 years | 5-10 years | ≥10 years |
| Coronary heart disease | no | ≤5 years | 5-10 years | ≥10 years |
| Coronary heart disease treatment | no | aspirin | multiple drugs | heart stent |
| Peptic ulcer | no | ≤5 years | 5-10 years | ≥10 years |
| Chronic liver disease | no | ≤5 years | 5-10 years | ≥10 years |
| Liver cirrhosis | no | ≤5 years | 5-10 years | ≥10 years |
| Cerebrovascular disease | no | ≤5 years | 5-10 years | ≥10 years |
| Other cancers | | | | |
| Nasopharynx cancer | no |  |  | yes |
| Laryngeal cancer | no |  |  | yes |
| Gastric cancer | no |  |  | yes |
| Liver cancer | no |  |  | yes |
| Pancreatic cancer | no |  |  | yes |
| Esophagus cancer | no |  |  | yes |
| Thyroid cancer | no |  |  | yes |
| Breast cancer | no |  |  | yes |
| Colorectal cancer | no |  |  | yes |
| Renal cancer | no |  |  | yes |
| Cerebral cancer | no |  |  | yes |
| Prostatic cancer | no |  |  | yes |
| Testicular cancer | no |  |  | yes |
| Ovarian cancer | no |  |  | yes |
| Cervical cancer | no |  |  | yes |
| Uterus Cancer | no |  |  | yes |
| Metastasis | | | | |
| Brain metastases | no |  |  | yes |
| Osseous metastasis | no |  |  | yes |
| Hepatic metastases | no |  |  | yes |
| Adrenal metastasis | no |  |  | yes |
| Renal metastasis | no |  |  | yes |
| Chest metastasis | no |  |  | yes |
| Pleural metastasis | no |  |  | yes |
| Skull metastasis | no |  |  | yes |
| Lymphatic metastasis | no |  |  | yes |
| Personal history | | | | |
| Smoking | never | cessation | occasionally | everday |
| Pack-years of smoking | 0 | <20 | 20-40 | ≥40 |
| Drink | never | cessation | occasionally | everday |
| Pack-years of drinking | 0 | <20 | 20-40 | ≥40 |
| Drug allergy history | no |  |  | yes |
| Operation History | no |  |  | yes |
| Family history | no |  |  | yes |
| Physical examination | | | | |
| Breathe(Times/minutes) | 12-20 | 9-11 | 21-24 | ≤8 or ≥25 |
| Temperature(℃) | 36.1-37.3 | 35.1-36.0 or 37.4 -39.0 | ≥39.1 | ≤35.0 or ＞41 |
| Heart rate(Times/minutes) | 60-90 | 41-59 or 91-110 | 111-130 | ≤40 or ≥131 |
| Systolic pressure(mmHg) | 110-130 | 131-139 or  101-109 | 140-170 or  90-100 | >170 or <90 |
| Diastolic pressure(mmHg) | 60-80 | 80-89 | ≥90 |  |
| General condition | | | | |
| Mental condition | no | slight | medium | severe |
| Nutritional state | no | slight | medium | severe |
| Physical development | no | slight | medium | severe |
| Face | no | slight | medium | severe |
| Hair | no | slight | medium | severe |
| Skin | no | slight | medium | severe |
| Superficial lymph node | no | slight | medium | severe |
| Eyesight | no | slight | medium | severe |
| Skull deformity | no | slight | medium | severe |
| Hearing | no | slight | medium | severe |
| Hoarseness | no | slight | medium | severe |
| Cyanosis | no | slight | medium | severe |
| Dental ulcer | no | slight | medium | severe |
| Aphasia | no | slight | medium | severe |
| Distention of jugular vein | no | slight | medium | severe |
| Thoracocyllosis | normal |  |  | abnormal |
| Intercostal change | normal |  |  | abnormal |
| Three depressions sign | normal |  |  | abnormal |
| Sense pleural friction | normal |  |  | abnormal |
| Moist rales | no | slight | medium | severe |
| Wheezes | no | slight | medium | severe |
| Velcro | no | slight | medium | severe |
| Voice transmission | no | slight | medium | severe |
| Cardiac souffle | no | slight | medium | severe |
| Pericardial friction rub | no |  |  | yes |
| Peripheral vascular examination | normal |  |  | abnormal |
| Abdominal mass | no |  |  | yes |
| Abdominal respiration | no |  |  | yes |
| Abdominal varicose veins | no |  |  | yes |
| Abdominal tenderness | no |  |  | yes |
| Murphy sign | no |  |  | yes |
| Hepatomegaly | no |  |  | yes |
| Splenomegaly | no |  |  | yes |
| Bowel sound | no |  |  | yes |
| Liver area knocking pain | no |  |  | yes |
| Renal area knocking pain | no |  |  | yes |
| Shifting dullness | no |  |  | yes |
| Ataxia | no |  |  | yes |
| Clubbing finger | no |  |  | yes |
| Ankylosis | no | slight | medium | severe |
| Redness and swelling of joints | no | slight | medium | severe |
| Joint tenderness | no | slight | medium | severe |
| Limb deformities | no |  |  | yes |
| Amyotrophy | no |  |  | yes |
| Lower limb edema | no | slight | medium | severe |
| Anaesthesia | no | slight | medium | severe |
| Muscular tension | normal |  |  | abnormal |
| Corneal reflex | normal |  |  | abnormal |
| Abdominal reflexes | normal |  |  | abnormal |
| Tendon reflex | normal |  |  | abnormal |
| Patellar tendon reflex | normal |  |  | abnormal |
| Achilles tendon reflex | normal |  |  | abnormal |
| Hoffmann sign | normal |  |  | abnormal |
| Babinski sign | normal |  |  | abnormal |
| Oppenheim sign | normal |  |  | abnormal |
| Kernig sign | normal |  |  | abnormal |
| Brudzinski sign | normal |  |  | abnormal |
| Pathological examination | | | | |
| Pathology grade(WHO) | no | I | II | III |
| Maximum tumor diameter | no | >2 | 2-4 | >4 |
| Differentiation | precancerous lesions | Well differentiated | moderately differentiated | poorly differentiated |
| Primary site | no | 1 | 2 | >2 |
| Number of primary lesions | no | 1 | 2 | >2 |
| Number of lymph nodes involved | no | 1-2 | 3-5 | >5 |
| EBER | negative |  |  | positive |
| EMA | negative |  |  | positive |
| ER | negative |  |  | positive |
| PR | negative |  |  | positive |
| HER-2 | negative |  |  | positive |
| GFAP | negative |  |  | positive |
| Olig-2 | negative |  |  | positive |
| BRAF | negative |  |  | positive |
| K-ras | negative |  |  | positive |
| Vimentin(VIM) | negative |  |  | positive |
| S-100 | negative |  |  | positive |
| D2-40 | negative |  |  | positive |
| CD34 | negative |  |  | positive |
| CD56 | negative |  |  | positive |
| CDX2 | negative |  |  | positive |
| Cam5.2 | negative |  |  | positive |
| CgA | negative |  |  | positive |
| P53 | negative |  |  | positive |
| MC | negative |  |  | positive |
| NapsinA | negative |  |  | positive |
| NF | negative |  |  | positive |
| calretinin | negative |  |  | positive |
| β-catenmin | negative |  |  | positive |
| cyclin D1 | negative |  |  | positive |
| IDH1 | negative |  |  | positive |
| SOX10 | negative |  |  | positive |
| Syn | negative |  |  | positive |
| PTEN | negative |  |  | positive |
| PD-1 | negative |  |  | positive |
| PD-L1 | negative |  |  | positive |
| EGFR | negative |  |  | positive |
| T790 | negative |  |  | positive |
| ALK | negative |  |  | positive |
| ROS | negative |  |  | positive |
| CK | negative |  |  | positive |
| CK5/6 | negative |  |  | positive |
| CK7 | negative |  |  | positive |
| CK8/18 | negative |  |  | positive |
| CK14 | negative |  |  | positive |
| CK20 | negative |  |  | positive |
| P63 | negative |  |  | positive |
| P40 | negative |  |  | positive |
| LCA | negative |  |  | positive |
| Ki-67 | negative |  |  | positive |
| T(tumor) | Tx and T1 | T2 | T3 | T4 |
| N(lymph node) | no | N1 | N2 | N3 |
| Metastasis | Mx and M0 |  |  | M1 |
| Imageological examination | | | | |
| Heart | normal |  |  | abnormal |
| Pericardium | normal |  |  | abnormal |
| Lung | normal |  |  | abnormal |
| Mass number | 0 | 1 | 2 | >2 |
| Tumor diameter | no | <1cm | 1-2cm | >2cm |
| Hilar | normal |  |  | abnormal |
| Mediastinum | normal |  |  | abnormal |
| Lymphadenectasis | 0 | 1-2 | 3-5 | >5 |
| Hydrothorax | 0 | <300ml | 300-500ml | >500ml |
| Emphysema | no |  |  | yes |
| Lung texture change | normal |  |  | abnormal |
| Active lesions | normal |  |  | abnormal |
| Old lung lesions | normal |  |  | abnormal |
| Electrocardiogram sign | | | | |
| Sinus rhythm | normal |  |  | abnormal |
| Conduction block | normal |  |  | abnormal |
| Atrial fibrillation | normal |  |  | abnormal |
| Ventricular premature contraction | normal |  |  | abnormal |
| Atrial premature contractions | normal |  |  | abnormal |
| P-wave | normal |  |  | abnormal |
| QRS | normal |  |  | abnormal |
| ST segment | normal |  |  | abnormal |
| T-wave | normal |  |  | abnormal |
| Q-T interval | normal |  |  | abnormal |
| Abdomen ultrasound | | | | |
| Liver | normal |  |  | abnormal |
| Pancreas | normal |  |  | abnormal |
| Spleen | normal |  |  | abnormal |
| Cholecyst | normal |  |  | abnormal |
| Adrenal gland | normal |  |  | abnormal |
| kidney | normal |  |  | abnormal |
| Ureter | normal |  |  | abnormal |
| Bladder | normal |  |  | abnormal |
| Abdominal lymph node | 0 | 1-2 | 3-5 | >5 |
| Lung CT | | | | |
| Trachea and bronchus | normal |  |  | abnormal |
| Pleura | normal |  |  | abnormal |
| Chest wall soft tissue | normal |  |  | abnormal |
| Coronary artery | normal |  |  | abnormal |
| Lung consolidation | normal |  |  | abnormal |
| Pulmonary embolism | normal |  |  | abnormal |
| Post enhancement | normal |  |  | abnormal |
| Lesions progress | normal |  |  | abnormal |
| Lung function | | | | |
| Restricted ventilation dysfunction | no | slight | medium | severe |
| Obstructive ventilation function disturbance | no | slight | medium | severe |
| Oxygen saturation(%) | no | slight | medium | severe |
| Vital capacity | no | slight | medium | severe |
| FEVI | no | slight | medium | severe |
| MEFV | no | slight | medium | severe |
| Cardiac ultrasound | | | | |
| EF (%) | 50-75 | 40-50 | 30-40 | <30 |
| E/E' | <8 | E/A>1 | E/A<1 | >15 |
| PASP(mmHg) | ≤30 | 30-40 | 40-50 | >50 |
| LVH | normal |  |  | abnormal |
| LVDF | normal |  |  | abnormal |
| Aortic regurgitation | no | slight | medium | severe |
| Pulmonary regurgitaion | no | slight | medium | severe |
| Calcification | no | slight | medium | severe |
| Head CT | | | | |
| Face | normal |  |  | abnormal |
| Maxillofacial | normal |  |  | abnormal |
| Neck | normal |  |  | abnormal |
| Pituitary gland | normal |  |  | abnormal |
| Frontal lobe | normal |  |  | abnormal |
| Parietal lobe | normal |  |  | abnormal |
| Temporal lobe | normal |  |  | abnormal |
| Occipital lobe | normal |  |  | abnormal |
| Pons | normal |  |  | abnormal |
| Cerebellar hemisphere | normal |  |  | abnormal |
| Sulci and gyri | normal |  |  | abnormal |
| Ventricle and cisterna lesions | normal |  |  | abnormal |
| Signal uniformity | normal |  |  | abnormal |
| Cerebral hernia | normal |  |  | abnormal |
| Focal ischemia | no | slight | medium | severe |
| MRI examination | | | | |
| Cervical | normal |  |  | abnormal |
| Thoracic | normal |  |  | abnormal |
| Lumber | normal |  |  | abnormal |
| Sacral | normal |  |  | abnormal |
| Neck 3-4 | normal |  |  | abnormal |
| Neck 4-5 | normal |  |  | abnormal |
| Neck 5-6 | normal |  |  | abnormal |
| Neck 6-7 | normal |  |  | abnormal |
| Lumbar 1-2 | normal |  |  | abnormal |
| Lumbar 2-3 | normal |  |  | abnormal |
| Lumbar 3-4 | normal |  |  | abnormal |
| Lumbar 4-5 | normal |  |  | abnormal |
| Lumbar 5-sacral 1 | normal |  |  | abnormal |
| Sacral 1-5 | normal |  |  | abnormal |
| Caudal 1-3 | normal |  |  | abnormal |
| Abdominal CT | | | | |
| Stomach | normal |  |  | abnormal |
| Perigastric lymph node | normal |  |  | abnormal |
| Intrahepatic bile duct | normal |  |  | abnormal |
| Pancreatic duct | normal |  |  | abnormal |
| Epityphlon | normal |  |  | abnormal |
| Intestinal wall | normal |  |  | abnormal |
| Enteric cavity | normal |  |  | abnormal |
| Seroperitoneum | normal |  |  | abnormal |
| Urine/stool routines | | | | |
| Urine color | normal |  |  | abnormal |
| Urine transparency | - | ± | + | ≥++ |
| Urine PH | 4.5-8.0 |  |  | <4.5 or >8.0 |
| Urine specific gravity | 1.000-1.030 |  |  | <1.000 or >1.030 |
| Urine glucose | - | ± | + | ≥++ |
| Urine protein | - | ± or + | ++ | >++ |
| Urobilirubin | - | ± | + | ≥++ |
| Urobilinogen | - | ± | + | ≥++ |
| Urine acetone bodies | ± | + | ++ | ≥+++ |
| Urinary nitrite | - | ± | + | ≥++ |
| Urine occult blood | - | ± | + | ≥++ |
| Urine leukocyte (/ul) | - | ± | + | ≥++ |
| Urinary vitamin C | - | ± | + | ≥++ |
| Microscopic examination of red blood cells /HP | 0-3 | >3 | >5 | >8 |
| Microscopic examination of white blood cell /HP | 0-5 | >5 | >10 | >25 |
| Microscopic examination of crystallization number | - | ± | + | ≥++ |
| Urine erythrocyte (/ul) | 0-10 | 10.1-50.0 | 50.1-100.0 | >100.0 |
| Urinary non-squamous epithelial cells (/ UL) | 0-2 | >2 | >6 | >10 |
| - Urinary squamous epithelial cells (/ul) | 0-5 | >5 | >15 | >25 |
| Transparent tube type (/ul) | 0-2 |  |  | >2 |
| - Pathological tube type (/ul) | 0-0.5 |  |  | >0.5 |
| Yeast (/ ul) | 0-3 |  |  | >3 |
| Stool color | normal |  |  | abnormal |
| Stool property | normal |  |  | abnormal |
| Mucus | normal |  |  | abnormal |
| Fecal occult blood | normal |  |  | abnormal |
| Chemical measurements | | | | |
| AFP (ng/ml) | 0-9 | <200 | > 200 | > 400 |
| AFP-L3/AFP(%) | 0-10 | <3 xULN | >3-5 xULN | >5 xULN |
| CEA (ng/ml) | 0-5 | <3 xULN | >3-5 xULN | >5 xULN |
| CA242 (U/ml) | 0-25 | <3 xULN | >3-5 xULN | >5 xULN |
| CA50 (U/ml) | 0-25 | <3 xULN | >3-5 xULN | >5 xULN |
| CA199 (U/ml) | 0-25 | <3 xULN | >3-5 xULN | >5 xULN |
| CA125 (U/ml) | 0-35 | <3 xULN | >3-5 xULN | >5 xULN |
| CA153 (U/ml) | 0-14 | <3 xULN | >3-5 xULN | >5 xULN |
| CA724 (U/L) | 0-6.9 | <3 xULN | >3-5 xULN | >5 xULN |
| SCCA (ng/ml) | 0-1.5 | <3 xULN | >3-5 xULN | >5 xULN |
| NSE (ng/ml) | 0-16.3 | <3 xULN | >3-5 xULN | >5 xULN |
| CYFRA21-1(ng/ml) | 0-3.3 | <3 xULN | >3-5 xULN | >5 xULN |
| PSA (ng/ml) | 0-4 | <3 xULN | >3-5 xULN | >5 xULN |
| FPSA/TPSA (%) | ＞0.26 |  |  | beyond normal limits |
| PAP (ng/ml) | 0-2 | <3 xULN | >3-5 xULN | >5 xULN |
| Ferritin (ng/ml) | 30-400 | <3 xULN | >3-5 xULN | >5 xULN |
| Thyroglobulin (ng/ml) | 3.5-77 | <2 xULN | >2 xULN | >5 xULN |
| Thyroglobulin antibody (IU/ml) | 0-115 | <2 xULN | >2 xULN | >5 xULN |
| Anti-thyroid peroxidase antibody (IU/ml) | 0-34 | <2 xULN | >2 xULN | >5 xULN |
| Thyrotropin receptor antibody (IU/L) | 0-1.75 | <2 xULN | >2 xULN | >5 xULN |
| T4 (nmol/L) | 66-181 | <2 xULN | >2 xULN | >5 xULN |
| fT4 (pmol/L) | 12.0-22 | <2 xULN | >2 xULN | >5 xULN |
| T3 (nmol/L) | 1.3-3.1 | <2 xULN | >2 xULN | >5 xULN |
| fT3 (pmol/L) | 3.1-6.8 | <2 xULN | >2 xULN | >5 xULN |
| TSH (mIU/L) | 0.27-4.2 | <2 xULN | >2 xULN | >5 xULN |
| IgG (g/L) | 7.0-16 | <2 xULN | >2 xULN | >5 xULN |
| IgG4 (g/L) | 0.03-2.01 | <2 xULN | >2 xULN | >5 xULN |
| IgA (g/L) | 0.7-4 | <2 xULN | >2 xULN | >5 xULN |
| IgM (g/L) | 0.4-2.3 | <2 xULN | >2 xULN | >5 xULN |
| IgE (IU/mL) | 0-100 | <2 xULN | >2 xULN | >5 xULN |
| Complement C3 (g/L) | 0.9-1.8 | <2 xULN | >2 xULN | >5 xULN |
| Complement C4 (g/L) | 0.1-0.4 | <2 xULN | >2 xULN | >5 xULN |
| Transferrin (g/L) | 2-3.6 | <2 xULN | >2 xULN | >5 xULN |
| Ceruloplasmin (g/L) | 0.2-0.6 | <2 xULN | >2 xULN | >5 xULN |
| PH | 7.35-7.45 | 7.29-7.36 or 7.46-7.49 | 7.2-7.3 or 7.5-7.6 | <7.2 or >7.6 |
| PaO2 (mmHg) | ≥90 | 60-90 | 40-60 | <40 |
| PaCO2 (mmHg) | 35-45 | 46-49 or 29-34 | 50-70 or 20-30 | <20 or >70 |
| SaO2 (%) | ≥95 | 86-94 | 75-85 | <75 |
| PT (s) | 11-17.8 | ULN-20.8 (ULN+3) | 20.9-30 | <9s or >30s |
| APTT(s) | 25.4-38.4 | ULN-44.9 or LLN-19.9 | - 1. or 16-20 | >60s or <16s |
| Fibrinogen (g/L) | 2.0-4 | <2 or >4 | <1.5 or 5.5 | <1 or >6 |
| TT(s) | 11-17.8 | 17.9-29.9 | 30-60 | >60s |
| AT-A (%) | 75.6-113.4 | <2 xULN | >2 xULN | >5 xULN |
| FDP (mg/L) | 0-5 | 4.9-14.9 | 15-40 | >40 |
| D-Dimer (mg/L) | 0-0.232 | >0.232 | >0.5 | >1 |
| INR | ≤ XULN | <3 | 3.0-3.5 | >3.5 |
| HBVe Ag | negative |  |  | positive |
| HBVe Ab | negative |  |  | positive |
| HBVs Ag | negative |  |  | positive |
| HBVs Ab | negative |  |  | positive |
| HBVc Ab | negative |  |  | positive |
| HBVc Ab IgM | negative |  |  | positive |
| HCV Ab IgG | negative |  |  | positive |
| HEV Ab IgG | negative |  |  | positive |
| HEV Ab IgM | negative |  |  | positive |
| HBV DNA copy number | negative |  |  | positive |
| Treponema pallidum specific antibody | negative |  |  | positive |
| TRUST | negative |  |  | positive |
| HPV | negative |  |  | positive |
| HIV | negative |  |  | positive |
| EB | negative |  |  | positive |
| HP | negative |  |  | positive |
| Total cholesterol (mmol/L) | 3-5.7 | 5.7-7 | 7-8 | >8 |
| Triglyceride (mmol/L) | 0-1.7 | 1.7-3.0 | 3.0-4.0 | >4 |
| HDL (mmol/L) | 1.03-2.07 | 0.91-2.07 |  | ≤0.91 |
| LDL (mmol/L) | ≤3.12 | 3.12-3.16 | 3.16-3.64 | >3.64 |
| Glycosylated hemoglobin (%) | 4-6 | 6-8 | 8-9 | >9 |
| Fasting blood-glucose (mmol/L) | 4.1-5.9 | 5.9-7 | 2.8-4.1 or 7-24.8 | <2.8 or >24.8 |
| Glomerular filtration rate (ml/min*1.73m2) | ≥90 | 60-89 | 30-59 | <30 |
| ALB | 1.2-2.4 |  | beyond normal limits |  |
| Albumin (g/L) | 35-55 or normal | <35 or >55 | <28 or >60 | <20 or >80 |
| Total protein (g/L) | 65-85 | <65 or >85 | <50 or >90 | <30 or >100 |
| ALT (U/L) | 9-50 | <9 or >50 | >200 | >500 |
| AST (U/L) | 15-40 | <15 or >40 | >200 | >500 |
| ALP(U/L) | 45-125 | < 2 xULN | >2 xULN | >5 xULN |
| Lactic dehydrogenase (U/L) | 120-250 | >250 or <120 | >500 | >1000 |
| Cystatin C(mg/L) | 0.6-1.3 | <0.6 or 1.3-2.63 | 2.64-4.90 | >4.9 |
| Gamma-GT (U/L) | 10-60 | < 2 xULN | >2 xULN | >5 xULN |
| Total bilirubin (umol/L) | <34.2 | 34.2-171 | 171-342 | >342 |
| Direct bilirubin (umol/L) | <3.4 | 3.4-17.1 | 17.1-34.2 | >34.2 |
| Urea (mmol/L) | 3.2-7.1 | 7.2-9 or <3.2 | 9-20 | >20 |
| Creatinine (umol/L) | 58-110 | <450 | <707 | >1500 |
| Uric Acid (umol/L) | ≤420 | >420 | >600 | >750 |
| β2-MG (mg/L) | 0-2.7 | < 2 xULN | >2 xULN | >5 xULN |
| Na(mmol/L) | 137-145 | 146-155,or125-136 | 155-160 or 120-124 | >160 or <120 |
| K(mmol/L) | 3.5-5.1 | 3.2-3.4 | 2.5-3.1/5.2-6.1 | <2.5 or >6.2 |
| Cl(mmol/L) | 98-107 | <98 or >107 | <95 or >115 | <90 or >120 |
| Ca(mmol/L) | 2.1-2.55 | 1.74-2.09 or 2.56-2.99 | 1.5-1.75 or 3-3.25 | <1.5 or >3.25 |
| P(mmol/L) | 0.85-1.51 | <0.85 or>1.51 | <0.5 or >1.75 | <0.3 or >3 |
| Mg(mmol/L) | 0.75-1.02 | <0.75 or >1.02 | <0.5 or >3 | <0.4 or >5 |
| Procalcitonin | 0-0.1 | 0.11-0.2 | >0.2 | >0.5 |
| Hemoglobin (g/L) | male: 120-160; female: 110-150 | male：91-119; female：91-109 or male：161-164; female：151-159 | 50-90/male：165-184; female：160-164 | <50 or male：>185，female：>165 |
| HCT(%) | 40-50 | 20-40 or 50-60 | 15-20 or 60-65 | <15 or >65 |
| MCV (fl) | 82-100 |  | <82 or >100 |  |
| MCH(pg) | 27.0-34.0 |  | <27.0 or >34.0 |  |
| RDW (%) | 10.0-15.0 |  | <10 or >15 |  |
| WBC (×10^9/L) | 3.5-9.5 | 9.51-20 or 1.5-3.51 | >20 or 1-1.49 | <1 or >30 |
| Neutrophil count (×10^9/L) | 1.8-6.3 | <1.8 or >6.3 | <1.5 or >8 | <0.5 or >8.5 |
| Neutrophil ratio (%) | 40-75 | >75 or <40 | <37.5 or >80 | <12.5 or >85 |
| Lymphocyte count (×10^9/L) | 1.1-3.2 | >1 xULN or <1 xLLN | >6 | >7.5 |
| Lymphocyte ratio (%) | 20-50/normal | >1 xULN or <1 xLLN | >60 | >75 |
| Eosinophil count (×10^9/L) | 0.02-0.52 | ＞0.52 or＜0.02 | >1.5 | >5 |
| Eosinophil ratio (%) | 0.4-8 | >1 xULN or <1 xLLN | >15 | >50 |
| Basophil count (×10^9/L) | 0-0.06 | >1 xULN or <1 xLLN | >0.2 | >2 |
| Basophil ratio (%) | 0-1 | >1 xULN or <1 xLLN | >2 | >20 |
| Monocyte number (×10^9/L) | 0.1-0.6 | >1 xULN or <1 xLLN | >1 | >10 |
| Monocyte ratio (%) | 3.0-10.0 | >1 xULN or <1 xLLN | >20 | >80 |
| Platelet (×10^9/L) | 125-350 | 50-126 or 351-449 | 20-49 or 450-799 | ≥800 or <20 |
| MPV(fl) | 6.5-12 |  | beyond normal limits |  |
| PCT(ml/L) | 0.11-0.28 |  | beyond normal limits |  |
| PDW (fl) | 12.0-18.0 |  | beyond normal limits |  |
| CRP(mg/L) | ≤10 | 10--30 | 30-90 | >90 |
| BNP (pg/ml) | 0-100 | 100-200 | 200-1000 | >1000 |
| CK-MB (ng/ml) | 0-3.7 | >3.7 | >6 | >10 |
| Myohemoglobin (ng/ml) | 11.6-73 | >73 | >80 | >100 |
| Troponin I (ng/ml) | 0-0.06 | >0.06 | >0.2 | >0.4 |
